# Correction of large jawbone defect in the mouse using immature osteoblast–like cells and a 3D polylactic acid scaffold

**DOI:** 10.1093/pnasnexus/pgac151

**Published:** 2022-08-18

**Authors:** Shigeto Suzuki, Venkata Suresh Venkataiah, Yoshio Yahata, Akira Kitagawa, Masahiko Inagaki, Mary M Njuguna, Risako Nozawa, Yusuke Kakiuchi, Masato Nakano, Keisuke Handa, Masahiro Yamada, Hiroshi Egusa, Masahiro Saito

**Affiliations:** Division of Operative Dentistry, Department of Ecological Dentistry, Graduate School of Dentistry, Tohoku University, Sendai, Miyagi 980-8575, Japan; Division of Operative Dentistry, Department of Ecological Dentistry, Graduate School of Dentistry, Tohoku University, Sendai, Miyagi 980-8575, Japan; Division of Operative Dentistry, Department of Ecological Dentistry, Graduate School of Dentistry, Tohoku University, Sendai, Miyagi 980-8575, Japan; Division of Operative Dentistry, Department of Ecological Dentistry, Graduate School of Dentistry, Tohoku University, Sendai, Miyagi 980-8575, Japan; OsteRenatos Ltd. Sendai Capital Tower 2F, 4-10-3 Central, Aoba-ku, Sendai, Miyagi 980-0021, Japan; National Institute of Advanced Industrial Science and Technology, 2266-98 Anagahora, Nagoya, Aichi 463-8560, Japan; Division of Operative Dentistry, Department of Ecological Dentistry, Graduate School of Dentistry, Tohoku University, Sendai, Miyagi 980-8575, Japan; Division of Operative Dentistry, Department of Ecological Dentistry, Graduate School of Dentistry, Tohoku University, Sendai, Miyagi 980-8575, Japan; Division of Operative Dentistry, Department of Ecological Dentistry, Graduate School of Dentistry, Tohoku University, Sendai, Miyagi 980-8575, Japan; Division of Operative Dentistry, Department of Ecological Dentistry, Graduate School of Dentistry, Tohoku University, Sendai, Miyagi 980-8575, Japan; Division of Operative Dentistry, Department of Ecological Dentistry, Graduate School of Dentistry, Tohoku University, Sendai, Miyagi 980-8575, Japan; Department of Oral Science, Division of Oral Biochemistry, Graduate School of Dentistry, Kanagawa Dental University, Yokosuka, Kanagawa 238-8580, Japan; Division of Molecular and Regenerative Prosthodontics, Graduate School of Dentistry, Tohoku University, Sendai, Miyagi 980-8575, Japan; Division of Molecular and Regenerative Prosthodontics, Graduate School of Dentistry, Tohoku University, Sendai, Miyagi 980-8575, Japan; Division of Operative Dentistry, Department of Ecological Dentistry, Graduate School of Dentistry, Tohoku University, Sendai, Miyagi 980-8575, Japan; OsteRenatos Ltd. Sendai Capital Tower 2F, 4-10-3 Central, Aoba-ku, Sendai, Miyagi 980-0021, Japan

**Keywords:** human alveolar osteoblasts, mice calvaria osteoblasts, bone regeneration, polylactic acid scaffold, functional bone

## Abstract

Bone tissue engineering has been developed using a combination of mesenchymal stem cells (MSCs) and calcium phosphate–based scaffolds. However, these complexes cannot regenerate large jawbone defects. To overcome this limitation of MSCs and ceramic scaffolds, a novel bone regeneration technology must be developed using cells possessing high bone forming ability and a scaffold that provides space for vertical bone augmentation. To approach this problem in our study, we developed alveolar bone–derived immature osteoblast–like cells (HAOBs), which have the bone regenerative capacity to correct a large bone defect when used as a grafting material in combination with polylactic acid fibers that organize the 3D structure and increase the strength of the scaffold material (3DPL). HAOB-3DPL constructs could not regenerate bone via xenogeneic transplantation in a micromini pig alveolar bone defect model. However, the autogenic transplantation of mouse calvaria–derived immature osteoblast–like cells (MCOBs) isolated using the identical protocol for HAOBs and mixed with 3DPL scaffolds successfully regenerated the bone in a large jawbone defect mouse model, compared to the 3DPL scaffold alone. Nanoindentation analysis indicated that the regenerated bone had a similar micromechanical strength to native bone. In addition, this MCOB-3DPL regenerated bone possesses osseointegration ability wherein a direct structural connection is established with the titanium implant surface. Hence, a complex formed between a 3DPL scaffold and immature osteoblast–like cells such as MCOBs represents a novel bone tissue engineering approach that enables the formation of vertical bone with the micromechanical properties required to treat large bone defects.

Significance Statement Large bone defects that cannot heal spontaneously are increasing in association with an aging society, and the inability to adequately treat these defects represents an unmet medical need. This study shows that a complex of osteogenic cells, such as immature osteoblast like cells, and a polylactic-acid-polymer–based on 3D biodegradable scaffolds has the potential to overcome this drawback. These complexes were found to successfully induce bone regeneration in a vertical direction upon autologous transplantation into mouse jawbone defects and possess the micromechanical properties of native bone and are suitable for prosthetic rehabilitation, such as dental implantation. These complexes of immature osteoblasts and 3D scaffolding represent a novel bone regeneration material for the treatment of large alveolar bone defects.

## Introduction

With recent advances in bone tissue engineering technology, the availability of bone substitute materials such as carbonate apatite, demineralized bovine bone, beta-tricalcium phosphate (β-TCP), and hydroxyapatite has reduced the burden on patients with regard to autologous transplantation, which requires additional surgical invasion for bone collection. Notably however, most of these materials are granular-type compounds that are only effective in treating mild bone defects surrounded by existing native bone (1–5). Three-dimensional bone substitute materials combined with a fibrillar matrix such as collagen or polymers have thus been developed for more complicated defects. Materials of this type containing a porous body, mainly comprising collagen and octacalcium phosphate, have been shown to be effective for bone forming treatments prior to implant placement, including maxillary sinus floor fistula surgery and tooth extraction socket preservation therapy, but have not been effective for large jawbone defects (6, 7).

Regeneration therapies for large bone defects will require the capacity to rapidly form bone in the vertical dimension. It has been shown previously that this type of growth can be achieved through a 3D arrangement of osteogenic cells, such as immature osteoblasts, in a scaffold. Human alveolar bone–derived immature osteoblasts (HAOBs) show higher osteogenic ability than mesenchymal stem cells (MSCs), can be expanded from the alveolar bone of patients older than 60 y, and can produce bone matrix in vivo without the need for treatment with osteogenic induction medium (8). HAOBs can be also obtained from alveolar bone during dental surgery by collagenase digestion (9, 10), differentiated into osteoblasts in vitro by exposure to osteogenic differentiation medium (ODM) and induce bone formation upon subcutaneous implantation into immunodeficient mice (8). A principal advantage of bone forming cells such as HAOBs is that they can reduce the surgical burden on patients undergoing autologous bone graft transplantation, which is the current standard treatment for large bone defects.

Horizontal bone defects are large jawbone defects that do not heal spontaneously because they require vertical bone augmentation, and this cannot be achieved efficiently with existing bone regeneration therapies (11). The number of patients who cannot receive dental implants due to large jawbone defects has increased dramatically in the past two decades and is expected to further expand in the future. The current standard treatment for these anomalies is autologous bone transplantation, which requires the collection of bone from the jaw or iliac crest, and its subsequent transplantation to bone defect site for more than 6 months to generate sufficient replacement bone, followed by implant therapy (12, 13). HAOBs are a candidate cell source for the treatment of large alveolar bone defects that require vertical bone augmentation. One of the critical requirements for successful regeneration of large bone defects is the development of a scaffold suitable for promoting osteoblast differentiation. Hence, the development of materials with appropriate stiffness that can promote osteogenic differentiation of HAOBs and withstand the mechanical load at transplantation sites is essential for this purpose (14).

In our present study, we describe the development of a 3D cotton-like scaffold structure produced by novel electrospinning technology using Poly laevorotatory Lactic Acid (PLLA) and gelatin (3DPL). This complex is suitable for HAOB differentiation to osteoblasts to regenerate large jawbone defects. We initially found that the xenotransplantation of HAOB-3DPL constructs, preconditioned in osteoblastic differentiation medium, did not promote bone formation in a micromini pig jawbone defect model. To resolve this problem, we here established a mouse alveolar bone defect model and investigated the bone regenerating ability of an autogenic transplantation of mice calvaria–derived osteoblasts (MCOBs), isolated using a protocol for HAOB preparations, and combined these cells with a 3DPL scaffold. We found using bone morphometric analysis that these MCOB-3DPL constructs regenerated new bone with suitable micromechanical properties for implant therapy. Our present study findings suggest that the autogenic transplantation of immature osteoblast–like cells and a 3DPL nanoarchitecture scaffold represents a viable new bone tissue engineering methodology for large jawbone defects.

## Results

### Design, fabrication, and characterization of 3D polylactic acid scaffolds

To achieve vertical bone augmentation, a 3D-polylactic acid scaffold (3DPL) was fabricated with high mechanical properties and a controlled scaffold microstructure to tailor the pore size and distribution, and the biodegradability rate, as illustrated in Fig. 1A to C. Adjustable 3DPL scaffolding was generated via electrospinning method using polylactic acid and gelatin. The fibers formed by electrospinning are similar to the collagen fibers of the extracellular matrix of bone and can be utilized as a porous base material for cell attachment and proliferation. In our present study, electrospinning is performed using polylactic acid and gelatin as raw materials (Fig. 1A).

**Fig. 1.**
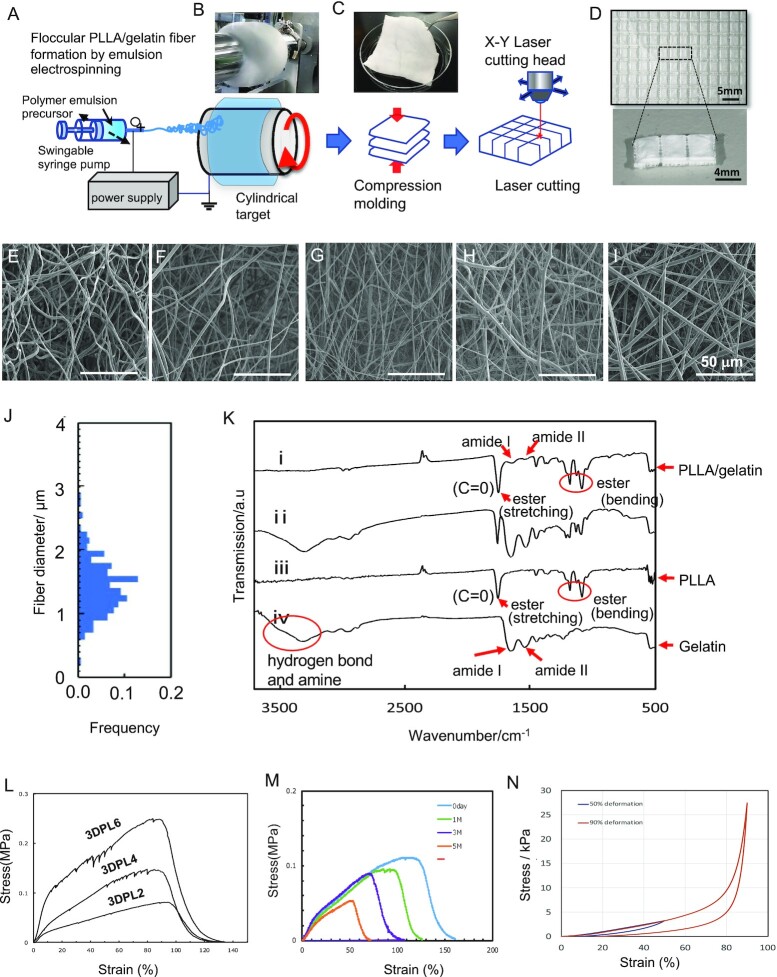
Synthesis of PLLA/gelatin floccular scaffolds for bone regeneration therapy. (A) Schematic illustration of the fabrication process used to produce PLLA/gelatin floccular scaffolds, based on PLLA/gelatin floccular fibers, using emulsion electrospinning. (B) Photograph of as-formed floccular PLLA/gelatin fabrics on the target electrode and (C) cut PLLA/gelatin floccular fabrics. (D) Photograph of the PLLA/gelatin floccular scaffolds. (E to I) SEM images of the PLLA/ gelatin floccular scaffolds (E, 3DPL1) (F, 3DPL2), (G, 3DPL4), (H, 3DPL6), and 2D sheet scaffolds (I, 2DPL). Scale bar, 50 μm. (J) Fiber diameter distributions of the PLLA/ gelatin floccular fibers from laser confocal microscopy (LCM) observations. (K) IR spectra of the PLLA/gelatin floccular fibers (i) and their chemically etched residue (ii) with IR spectra of the PLLA (iii) and gelatin (iv) included for comparison. (L) Tensile stress–strain curves of the floccular PLLA/gelatin nonwoven fabrics. (M) Degradation of the PLLA/ gelatin floccular scaffolds under a wet environment. (N) Compression stress–strain curves of floccular PLLA/gelatin nonwoven scaffolds (3DPL4).

PLLA/gelatin cotton-like fabrics (hereafter referred as PLA fabrics) were then formed by collection with a cylindrical target (Fig. 1B), compression processing of stacked PLA fabrics (8 × 8 cm^2^) was performed (Fig. 1C), and the size (length 5 mm × width 3 mm × height 2 mm) was adjusted by laser cutting to create the 3DPL scaffold material (Fig. 1D). The thickness of the 3DPL scaffold material was confirmed by vernier caliper (Fig. S1). The fiber diameter distribution of all the different lots of PLLA/gelatin floccular fibers was estimated from the LCM using the accompanying LCM analysis software (LEXT OLS application program, OLYMPUS, Japan) (Fig.S2). The X-axis represents the diameter of electrospun fibers, while the Y-axis contains the diameter distributions obtained by analyzing 180 fibers in each specimen. The average fibre diameter lies in the range of 1.2 μm. No differences in fiber diameter distribution were found in the beeswarm plots of the eight lots of PLL/gelatin floccular fibers (Fig. S2). Four kinds of 3DPL scaffolds (3DPL1, −2, −4, and −6) with varying degrees of mechanical stiffness were prepared by compression molding. Here, the number of 3DPL scaffold is the number of stacked PLA fabrics in compression process (for example: 3DPL4, when the number of stacked PLA fabrics is 4). Hence, the density of the scaffold fibers increases with higher stacked PLA fabrics rendering to increased stiffness, but the fiber diameter remains constant for all types of 3DPL scaffolds. The topology of the 3DPL1 (Fig. 1E), 3DPL2 (Fig. 1F), 3DPL 4 (Fig. 1G), and 3DPL 6 (Fig. 1H) scaffold surfaces was demonstrated by SEM analysis, which revealed a characteristic 3D morphology with randomly oriented polylactic (PL) fibers. By contrast, the 2D PL scaffold (2DPL), which has a nonwoven fabric structure, showed a linear fiber structure (Fig. 1I). The diameter of the fibers used for the 3DPL scaffolds was set to 1.5 μm to meet the requirements for bone tissue regeneration (Fig. 1J) and was confirmed by LCM. The chemical identity of the 3DPL scaffold was analyzed using Fourier-transformed infrared spectroscopy (FTIR). The FTIR spectrum in Fig 1K (iii) showed characteristic absorption bands at 1,750 cm^−1^ related to C = O (ester stretch) and a short bands at 1,180 and 1,080 cm^−1^ (ester bend), which represents the backbone ester group of PLLA. Fig 1K (iv), in addition to the hydrogen bond and amide as a peak at 3,500 and 3,300 cm^−1^, the amide I and II bands are seen as the peaks at 1,640 and 1,550 cm^−1^ are related to gelatin. These spectra indicated that the tested samples were typically made of PLLA/gelatin without the presence of significant contaminants (Fig. 1K). As explained above, four types of 3DPL scaffolds were prepared to assess their suitability for immature osteoblast attachment, proliferation, and differentiation. 3DPL1 and −6 showed the lowest and highest stiffness, respectively. The mechanical testing results for the panel of 3DPL scaffolds are shown in Fig. 1L. A significant increase in stiffness was observed as the fiber density increased in the 3DPL2, −4, and −6 scaffolds, respectively. These scaffolds were kept at 37°C under wet conditions for 1, 3, and 5 months, and the mechanical properties were deteriorated due to the hydrolysis of PLA. The mechanical strength was found to be maintained to the extent that it could be handled or shaped (Fig. 1M). In a compression test on a 1 cm^3^ test piece with a fiber density equivalent to that of 3DPL scaffolds, the elastic restoration of the scaffold shape was observed during by unloading even after a 90% compressive deformation, and the samples were not crushed. In that experiment, the restoration rate for the compression variant was about 80% (Fig. 1N).These overall structural parameters of the 3DPL scaffold enabled its use in cell seeding experiments for bone tissue engineering.

### Xenotransplantation of the HAOB-3DPL4 complex does not induce bone regeneration in the pig

The standard approach for bone tissue engineering involves a combination of osteogenic cells and a biocompatible scaffold material. We seeded a HAOB cell suspension onto our 3DPL4 scaffold to prepare a tissue-engineered construct and investigated its osteogenic characteristics in vitro and bone regeneration ability in vivo via transplantation into a pig alveolar bone defect model.

We first conducted in-vitro osteogenic gene expression analysis to identify the most suitable osteogenic stimulant for the HAOB-3DPL4 constructs. We developed a 28-day culture model that included an initial proliferation phase of 7 days in basal medium to allow HAOBs to proliferate and migrate within the scaffold, followed by a 14- and 21-days induction with osteogenic stimulants (Fig. 2A). The results of Q-PCR analysis provided evidence of osteogenic differentiation by detecting the expression of OSTERIX, RUNX2, TYPE I COLLAGEN, and BONE SIALOPROTEIN (BSP) at both 14 (Fig. 2B) and 21 (Fig. 2C) days. No significant difference was observed in osteogenic gene expression by HAOB treated with the SAG, TH, SAG+TH, and BMP2 groups at 14 days of incubation. However, TYPE I COLLAGEN was significantly decreased by HAOB treatment with BMP2 and SAG compared to TH and SAG+TH at 21 days.

**Fig. 2.**
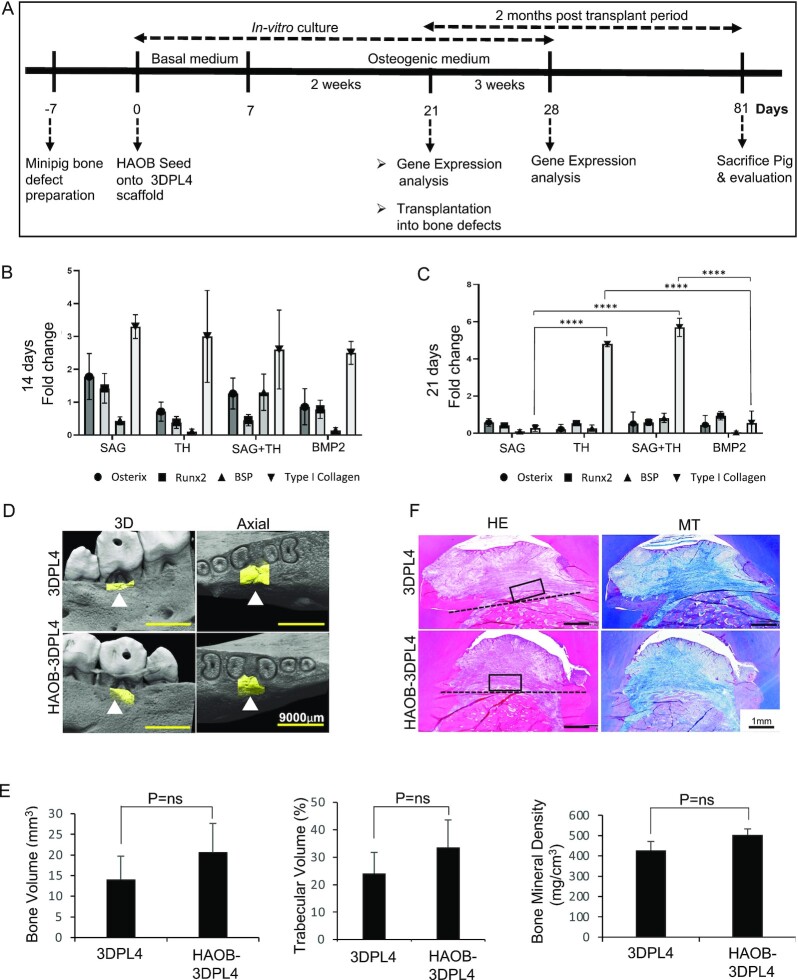
HAOB-3DPL4 constructs fail to regenerate bone following xenotransplantation into a micromini pig furcation defect model. (A) Overview of the timeline for defect preparation and HAOB-3DPL4 scaffold construct preparation for in-vitro osteogenic characterization and transplantation into a bone defect. (B, C) Osteogenic-related gene expression of HAOB-3DPL4 scaffold constructs after 14- and 21-days incubation under osteogenic medium. (D) Three dimensional reconstruction of micro-CT images and axial view of the furcation defect following HAOB-3DPL4 constructs and 3DPL4 scaffold transplantation into the established micromini pig furcation defect model. (E) Quantification of the regenerated alveolar bone in terms of bone volume, trabecular bone, and bone mineral density from reconstructed 3D micro-CT images. Representative sections of 3D4PL4 and HAOB-3D4PL4 scaffold constructs examined by hematoxylin–eosin (HE) and Masson’s trichrome (MT) staining. (ns; not significant). (F) Representative sections of HAOB-3DPL4 constructs and 3DPL4 scaffold transplant samples were examined by Haematoxylin and Eosin (H&E) staining.

We next investigated the in-vivo bone regenerative ability of HAOB-3DPL4 constructs following their transplantation into a micromini pig alveolar bone defect model. Defects filled with scaffold alone served as a control group. Healing progressed uneventfully without an intense inflammatory reaction during the 8-week observation period. The animals were sacrificed at 8-week post-transplantation and the jaws were collected to investigate new bone formation by μCT and histological analysis. Three dimensional reconstructions of the μCT images revealed new alveolar bone formation (Fig. 2D, yellow region) in both the HAOB-3DPL4 and 3DPL4 (control) groups. Quantitative bone volume analysis of the defect area indicated no significant differences in the bone volume, trabecular volume, and bone mineral density between the control and HAOB-3DPL4 groups (Fig. 2E). We next performed histological analysis to evaluate new bone regeneration. The experimental area was sectioned and stained with HE and MT. The resulting HE and MT-stained photomicrographs indicated minimal bone formation above the base of the defect (outlined by dotted line) in both the control and HAOB-3DPL4 groups (Fig. 2F). A possible reason the lack of any significant new bone regeneration in the HAOB-3DPL4 group could be a rejection of the HAOB cells by the mini pig host immune system.

### The autologous transplantation of MCOB-3DPL4 constructs successfully induces bone regeneration in a mouse alveolar bone defect model

We next investigated the bone regenerative ability of mouse calvaria–derived MCOBs that had been collected in the same way as HAOBs and combined with the 3DPL4 scaffolds (Fig. S6). These constructs were then transplanted into large alveolar bone defects in a mouse model. We first investigated the in-vitro osteogenic ability of the MCOBs when cultured in ODM at 10-day incubation. Under ODM treatment, exhibited strong ALP activity, as indicated by positive violet staining, and intense staining of calcified nodules by alizarin red (Fig. 3A, lower panel). MCOBs without ODM induction displayed weak ALP and alizarin staining, indicating their undifferentiated state (Fig. 3A, upper panel). Quantitative PCR analysis revealed that osteogenic genes including osterix, osteocalcin, and runx2 were more highly expressed in the MCOBs grown in ODM, further indicating their osteogenic differentiation ability (Fig. 3B). The adhesion of the required bone cells to a scaffold is an important prerequisite for successful bone tissue engineering. Following confirmation of the osteogenic ability of MCOBs, we investigated their attachment capacity to the 3DPL4 scaffold by SEM analysis 2 hours after seeding (Fig. 3C, left). High magnification analysis of these constructs showed that the majority of the attached cells were round in shape with cell–cell and cell–3DPL4 contact, indicated by red arrows and that PL fibers were barely visible (Fig. 3C, right). SEM analysis of the 3DPL4 scaffold without cells (Fig. 3D, left) clearly showed the presence of only PL fibers at high magnification (Fig. 3D, right). To then investigate the in-vivo bone forming ability of the MCOB-3DPL4 constructs, we performed subcutaneous implantation into C57BL/6N mice as a model of autologous transplantation. H&E staining of transplanted scaffold constructs seeded with MCOBs at both 4- and 8-weeks post-transplantation indicated that bone-like tissue formation had occurred within the 3DPL4 fibers (Fig. 3E, upper and lower panel). In contrast, the 3DPL4 scaffolds without cells showed only connective tissue formation (Fig. 3F, upper and lower panel). These data confirmed that MCOB-3DPL4 scaffold constructs are osteoinductive and that MCOBs seeded within the scaffold contribute to new bone tissue formation. To further verify the jawbone regeneration ability of the MCOB-3DPL4 constructs, we investigated whether they promoted the regeneration of functional bone in a maxillary bone defect mouse model (Fig. S6, Movie S1). Two dimensional μCT images of the MCOB-3DPL4 transplant group confirmed bone regeneration in the defect area, as indicated by increased radiodensity at both 4- and 8-weeks post-transplantation, reaching up to the cemento-enamel junction (CEJ) (Fig. 4A, Fig. S8A and B). A control group in which the defect was filled with cytrans, a calcium carbonate–based bone substitutes approved for clinical applications, showed a comparable level of new bone formation. However, the increased radiodensity in the cytrans group was partially due to retained carbonate apatite granules. In the 3DPL4 and empty defect comparison groups, a minimal amount of bone formation was observed. Quantification of the new bone formation in the defect area indicated no significant difference between any of the experimental groups at 4 weeks (Fig. 4B, left panel), but a significantly higher level in the MCOB-3DPL4 and cytrans groups compared to the empty and 3DPL4 groups at 8-weeks post-transplantation (Fig. 4B, right panel), and no significant difference between the MCOB-3DPL4 and cytrans groups at this timepoint. From the 3D-reconstructed μCT images, buccal bone defects were clearly observed in the 3DPL4 and empty defect animals but completely recovered in the MCOB-3DPL4 and cytrans groups. Notably however, scattered, and residual carbonated apatite granules were observed in the cytrans group (Fig. 4C and Fig. S8B).

**Fig. 3.**
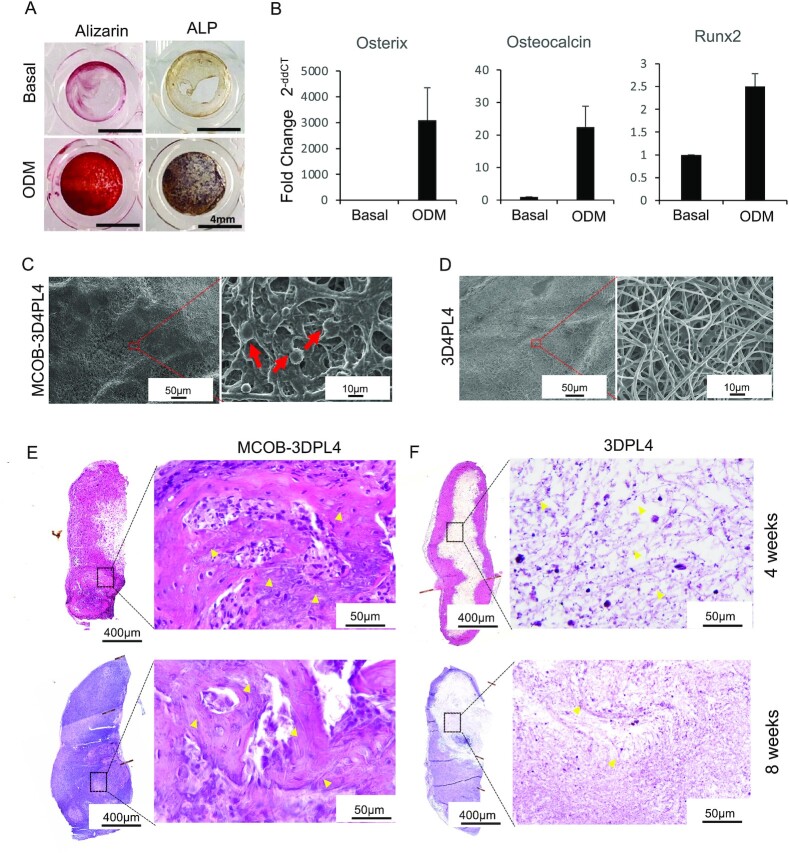
Analysis of the osteoblast differentiation ability of MCOB. (A) Representative images of alizarin red and ALP staining of MCOB at day 10 without (upper panel) and with (lower panel) ODM treatment. Relative positive alizarin and ALP activity detected only in MCOB conditioned with ODM. (B) The relative mRNA expression levels of Osterix, Osteocalcin, and Runx2 of MCOB treated with ODM for 10 days were shown as fold change by real time-PCR analysis. (C) SEM images of MCOB seeded in 3DPL4 scaffold (left). Higher magnification (right) showing external cell–cell and cell–3DPL4 contact indicated by red arrows. (D) SEM images of 3DPL4 scaffold (left). Higher magnification (right) showing 3D arrangement of polylactic acid fibers. (E) The photographs of H&E staining of MCOB-3DPL4 and 3DPL4 (F) implant groups at 4 weeks (upper) and 8 weeks (lower) after the subcutaneous transplantation into mouse. Boxed areas are shown at higher magnification. Higher magnified view of the MCOB-3DPL4 groups showed ectopic bone formation within 3DPL4 scaffold at both 4- and 8-weeks post-implantation, highlighted by yellow arrow heads. Highly magnified view of the 3DPL4 groups showed connective formation within 3DPL4 scaffold and yellow arrow heads indicate the PL fibers.

**Fig. 4.**
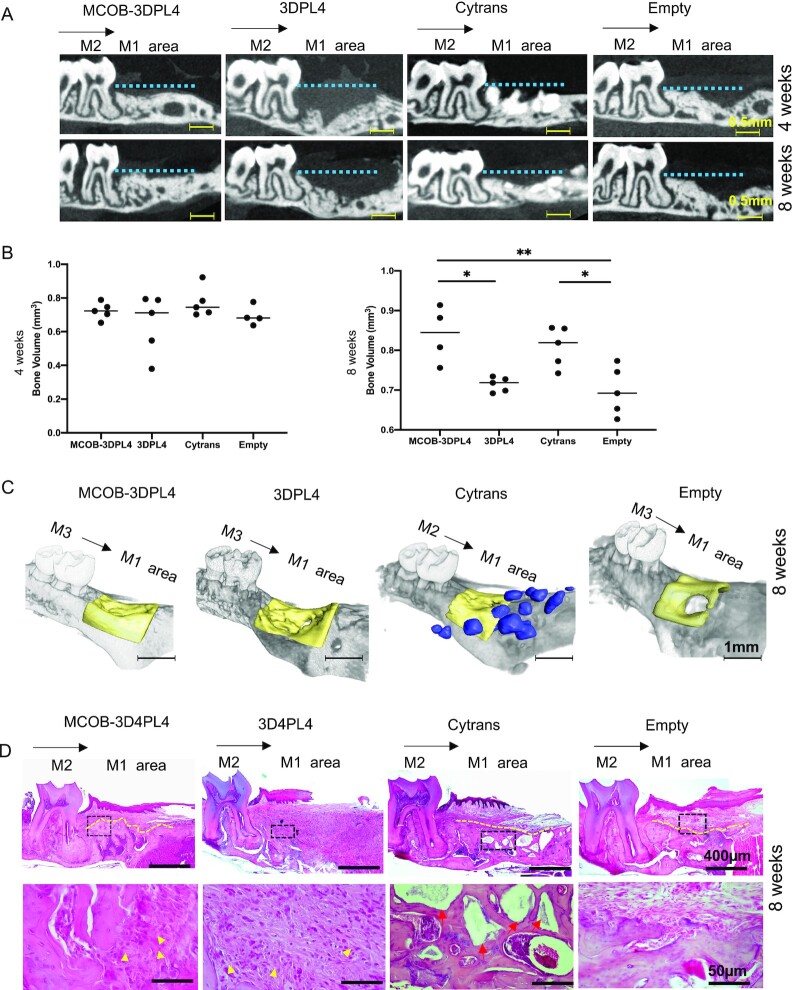
Bone regeneration capacity in the mice alveolar bone defect evaluated by microCT (μCT) and histological analysis. (A) Two dimensional μCT images of the defect areas, filled with MCOB-3DPL4, 3DPL4, Cytrans, and empty defect groups at 4- and 8-week post-transplantation. (B) μCT quantification of new bone volumes in the mice alveolar defect areas. (C) 3D-reconstructed images of the defect area. (D) Representative H&E–stained images of the low (Upper row) and high (framed regions of upper row) magnifications of the defect areas at 8-week post-transplantation. The yellow arrow heads indicate 3DPL4 fibers, red arrow heads indicate cytrans granules, and the M3: maxillary third molar, M2: maxillary second molar, and M1 area: maxillary first molar area/defect areas, black arrow indicates the mesial direction of the jaw, yellow-dotted line represents the outer edge of the regenerated bone, blue-dotted line represents the CEJ line, and the area below it indicates bone defect area.

### Histological analysis of the regenerated bone induced by MCOB-3DPLA constructs

To histologically investigate the regeneration of bone induced by MCOB-3DPLA, H&E staining was performed within the bone defect area in the mouse model. M1 (maxillary first molar) area in the all the histological images represents the bone defect area. (Fig. 4D and Fig. S9). In the MCOB-3DPL4 transplant group, nearly half of the defect area was filled with new bone at 4 weeks (Fig. S9) and mature, organized bone tissue along with bone marrow was formed at 8-weeks (Fig. 4D) post-transplantation. At higher magnification observations in the MCOB-3DPL4 group, new bone formation was found to be incorporated around 3DPL fibers (arrowheads) at 8-weeks post-transplantation (Fig. 4D, lower row). In the cytrans control group, partial new bone formation was observable around the residual carbonate apatite granules within the defect area at both 4- and 8-weeks post-transplantation (Fig. 4D and Fig. S9). The 3DPL4 control group showed the presence of inflammatory infiltrates in the defect area, while the empty group showed only partial granulation tissue and bone formation in the center of the defect area, at 4- and 8-weeks post-transplantation. (Fig. 4D and Fig. S9). These histological findings were thus consistent with the μCT data, both of which demonstrated a high amount of bone formation in the MCOB-3DPL4 and cytrans groups.

Overall, these data indicate that the defect areas transplanted with MCOB-3DPL4 and cytrans material showed active remodeling processes in the newly regenerated bone.

### Biomechanical properties of the newly regenerated bone

We next investigated the micromechanical properties of the regenerated bone in the mouse maxilla defect model using nanoindentation measurements, an effective technique for assessing the hardness of bone tissue (Fig. S11).

The micromechanical properties of the regenerated bone from the cytrans granules showed a greater ability to resist elastic deformation (Fig. 5A and B) due to retained residual carbonate apatite granules. The highest tissue hardness in the regenerated bone was observed in the cytrans group, followed by the 3DPL4 (represents native bone), MCOB-3DPL4, and empty defect groups. However, the hardness of the newly formed bone tissue was not significantly different between the experimental groups (Fig. 5A). The elastic modulus was also higher in the regenerated bone of the cytrans group compared to 3DPL4 and empty defect groups, but this difference between the cytrans and the MCOB-3DPL4 groups was also not significant (Fig. 5B). However, elastic modulus of the MCOB-3DPL4 group is comparable to the 3DPL4 (represents native bone) and empty groups. Overall, these data indicated that the regenerated bone in the MCOB-3DPL4 group possessed similar micromechanical properties to the native bone of the maxilla. The osseointegration ability of the regenerated bone was next evaluated by placement of a dental implant to assess the tolerance to implant therapy (Movie S2). The implants were placed in the area of regenerated bone (M1 area) in all of the experimental groups and osseointegration was tested after 4 weeks (Fig. S14) by μCT and histological analysis. The implant dropout ratio (remaining implants/implantation number) was 5/5 in both the MCOB-3DPL4 and 3DPL4 groups, which was superior to 3/5 in the control (empty) group and 4/5 in the cytrans group. Two dimensional μCT analyses (Fig. 5C) and 3D reconstructions (Fig. 5D) revealed that there was no complete osseointegration of the implants within the regenerated bone in any of the groups. We evaluated this further by H&E staining and found evidence of good osseointegration in the MCOB-3DPL4 and cytrans groups, and partial osseointegration in the 3DPL4 and empty defect groups (Fig. 5E).

**Fig. 5.**
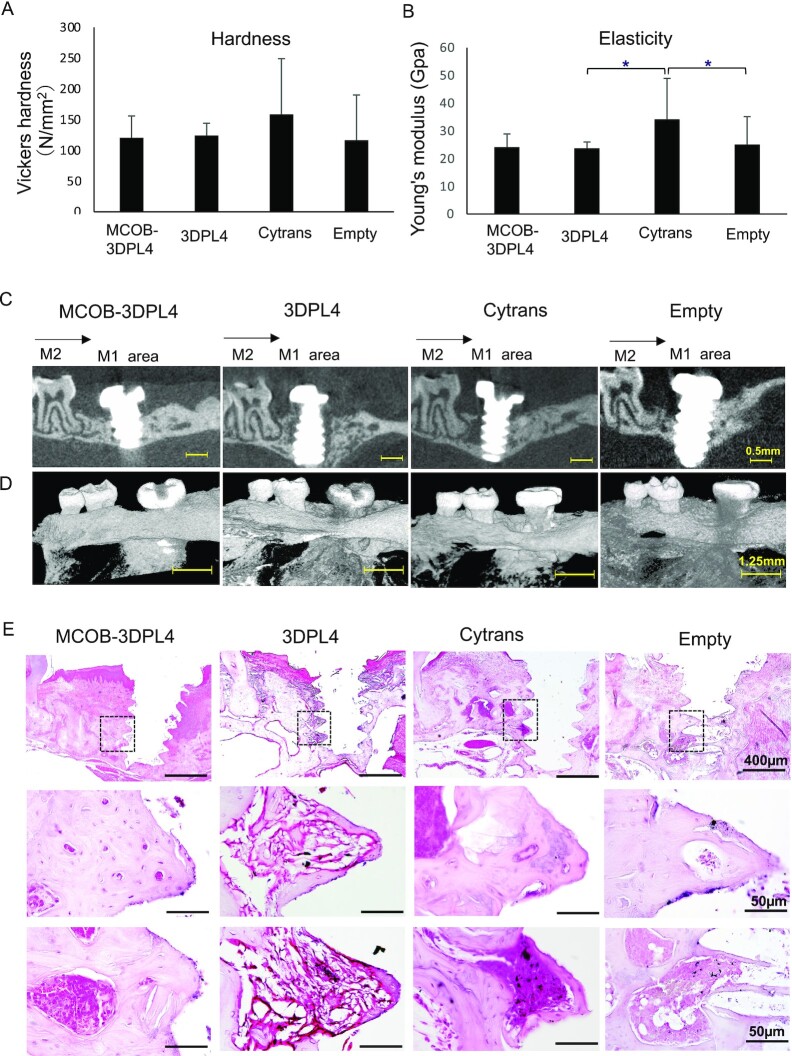
Biomechanical and functional properties of regenerated bone at 8-week post-transplantation. (A) Hardness and (B) elastic modulus of regenerated bone at a maximum load. (C) Two dimensional and (D) 3D μCT images of a mouse alveolar defect area at 4-week post-implant placement. (E) H&E staining of the mouse alveolar bone defect at 4 week after implant placement. Boxed regions are shown as highly magnified sections in the middle and lower rows. M2: maxillary second molar, M1 area: maxillary first molar area/regenerated bone area, and black arrow indicates mesial direction of the jaw.

## Discussion

New bone tissue engineering technologies have been developed to regenerate large bone defects that can adequately recapitulate bone developmental processes (15). We demonstrate in our present study that 3DPL4, a polylactic-acid–based nanoscale architecture scaffold, has the appropriate degree of mechanical strength to enable osteoblast differentiation and provide a suitable microenvironment for bone formation upon autologous transplantation of MCOBs into a large alveolar bone defect in the mouse maxilla. In addition, the MCOB-3DPL4 constructs were found to support the regeneration of bone in a vertical direction with sufficient mechanical strength for implant placement.

Macroscale architecture scaffolds have been developed previously for bone regeneration, mainly using calcium phosphate–based scaffolds (16–19). An essential characteristic of a macro porous calcium phosphate–based scaffold is the use of a combination of collagen and apatite to promote the attachment of cells with a flattened shape (20). In contrast to calcium phosphate-based materials, more recent nanofiber technologies have enabled the generation of nanoarchitecture scaffolds with pore sizes that are smaller than a cell diameter, which provides a 3D morphology that mimics the cells within connective tissues such as bone (21–24). The mechanical properties of the materials used in scaffolds influence the ability of cells to differentiate, and scaffolds of sufficient mechanical strength are required at the transplanted site for successful regeneration therapy (25). The electrospinning technique is a technology in which a high voltage is applied to a polymer solution to stretch the fibers with the electrostatic force of an electric field in the order of micro to nanometers (26–29). In conventional electrospinning, the formed electrospun fibers are strongly aggregated on the electrode and a 2D sheet-like nonwoven fabric is produced (30). However, these structures cannot provide a sufficient space for cell proliferation and are not suitable as a bone replacement material. To solve this problem, 3DPL was developed with intercommunicating pores that have characteristics of a fibrous porous body, which can withstand compression with 90% deformation, have an easily adjustable elastic modulus by fiber density, and provide an environment suitable for the osteoblast differentiation of HAOBs and MCOBs. This outcome resulted from increasing the width of the PL fibers to maintain porosity and layering to maintain their strength. This outcome was a result of formation of cotton-like PLA fibers with appropriate fiber diameter using emulsion-electrospinning. Such cotton-like PLA fibers have an enough flexibility and a sufficient modulus of elasticity by themselves to hold the spaces among the fibers. As seen in the compression test of the 3DPL4 scaffold, such flexibility and resiliency of the cotton-like PLA fiber was maintained even after compression process for fiber density adjustment.

Our present results indicated that HAOBs synthesize an extracellular matrix on a 3DPL4 scaffold via osteoblast differentiation, but the subsequent xenotransplantation of these constructs into a pig bone defect model did not induce bone formation. This indicated that HAOBs differ from MSCs and cannot be xeno- or allo-transplanted due to a low immunological tolerance of the host animal. We thus conducted autogenic transplantation using MCOBs in a mouse maxillary bone defect model. Vertical bone regeneration was achieved in this system by engrafting MCOB-seeded 3DPL4 constructs without preconditioning for osteoblast differentiation. In the current regenerative medicine technology field, the transplantation of undifferentiated cells has been found to regenerate body tissue functions in certain settings. For example, undifferentiated cardiomyocytes derived from induced pluripotent stem cells (iPSCs) were reported to differentiate into cardiomyocytes that restored cardiac function following transplantation into a rat heart failure model (31). In addition, the transplantation of undifferentiated Langerhans islet *β* cells, derived from human embryonic stem cells (ESCs), into diabetic immunodeficient mice was found to successfully recover the blood glucose levels in a treatment model of type I diabetes (32). In the present study, we found that xenogeneic transplantation of HAOB-3DPL4 scaffold constructs within the minipig alveolar bone defect generated poor bone formation. No significant differences were observed compared to the 3DPL4 scaffold without HAOB. However, MCOB, which was isolated similar to that of HAOB, combined with the 3DPL4 scaffold generated new bone formation and was significantly higher than the 3DPL4 scaffold without MCOB. This kind of anomalous response between two animal models was mainly due to the type of transplantation, xenogeneic, and autologous. Interestingly, many studies show similar results regarding the efficacy of xenogeneic transplants. For example, although MSCs are anticipated to have immunosuppressive and immunomodulatory functions, they have shown conflicting results when used for bone regeneration therapeutics following xenogeneic transplantation across species (33–36). Our study demonstrated that xenogeneic transplantation of HAOB into the Pig bone defect model showed no new bone formation due to undesired immune response mounted by pig host immune cells and possibly leading to transplanted cells’ death. However, autologous transplantation of MCOB into the mouse defect model showed significantly higher bone formation due to the absence of immune response against transplanted cells, secretion, and mineralization of collagen matrix. The results of our study demonstrate that reduced new bone formation occurred in the xenogeneic transplant groups from micro-CT and histological findings, despite the absence of evidence of immune rejection. Our study assumed that rejection of HAOB contributed causally to the results found in our study. The current study was designed to focus specifically on the efficacy of cell-scaffold constructs in new bone regeneration and investigate the mechanical properties of the regenerated bone. However, survival of the transplanted cells is a major challenge and one of the important criteria determining the success or failure of treatment. Since autologous transplantation was performed in this study, MCOB did not reject immediately upon transplantation, but we do not know how long cells survived at the transplantation site. To address this problem, MCOB isolated from GFP-transgenic mice will be utilized for tracing cells following transplantation in our future study. Nevertheless, the results of the present study support the evidence that autologous transplantation of osteoblasts is superior in promoting new bone formation. Thus, this autologous approach should be supported and considered for future translational research in bone regeneration therapeutics.

PL scaffolds are biocompatible and therefore have a potentially broad range of applications in the medical and dental field, including bone tissue engineering. In a previous study, however, the transplantation of PL scaffold alone was reported to induce inflammation (37), which was suppressed by silver ions (38). Similarly, in this present study, a 3DPL scaffold alone induced inflammation when transplanted into our mouse model of maxillary bone defects. We therefore speculated that the addition of MCOBs may also suppress the 3DPL-induced inflammatory response during their differentiation into osteoblasts on this scaffold. From our results and previous findings, it will clearly be necessary to prevent 3DPL-induced inflammation in a real-world clinical setting.

The challenge for regenerative therapy in correcting large bone defects requires functional analysis to verify whether regenerated bone formed by tissue engineering can actually be used in a clinical application (39). In addition, mechanical properties of tissue engineering scaffolds are vital to ensure the long-term structural and functional viability of cells in both in vitro and in vivo (40). Human cells sense the mechanical properties of the extracellular matrix, making them responsive to the mechanical cues from the environment (41–43). Our in-vitro studies showed increased staining intensity of alizarin in scaffolds with higher stiffness (3DPL4 and 3DPL6) than with low stiffness (3DPL1, 3DPL2, and 3DPL3), indicating that stiffer scaffolds are more appropriate for cell adhesion, differentiation, secretion of extracellular matrix, and mineralization. This observation goes along with other studies, which describes the stiffness of the scaffold plays an essential role in cell adhesion and differentiation and is better suitable for bone regeneration applications. From a bone tissue regeneration perspective, scaffolds with high stiffness favor osteogenic differentiation of MSCs (40, 42). We therefore used 3DPL4 scaffolds for all further experiments. To determine whether the regenerated bone formed by MCOB-3DPL4 constructs induced functional bone formation that could withstand masticatory function, the regenerated bone was analyzed using a nanoindentation test that can measure bone strength and elastic modulus on a small scale, enabling the quantification of bone mechanical properties (44). The calcification levels affect the strength and elasticity of bone, and are indicative of bone maturation and an increased mineral content, indicating that mechanical strength is a regulatory factor in the bone remodeling processes that play an important role in bone formation and homeostasis (45, 46). A carbonate apatite material such as cytrans has been reported to activate bone metabolism and bone regeneration at an early stage, and clinical studies using this scaffold have indicated that implant therapy can be performed with the regenerated bone induced by maxillary sinus floor elevation (47). However, there are no reported studies on the functional evaluation, including the micromechanical strength of the regenerated bone, induced by the same material. Our current study findings indicated that the regenerated bone in the cytrans transplanted group had higher micromechanical strength than the native maxillary bone, suggesting that the bone micromechanical properties had been affected by remaining cytrans within the maxillary bone defect area. On the other hand, the regenerated bone formed in the MCOB-3DPL4 transplanted group had identical micromechanical strength to the bones within the control group and also the 3DPLA alone transplanted group, indicating that the regenerated bone induced by MCOB-3DPL4 had the same micromechanical property as native bone, and that the PL fibers which had been incorporated into the regenerated bone did not affect bone strength. Osseointegration refers to bone fusion at the interface between dental implants and bone (48, 49) and has been observed in regenerated bone induced by cytrans. In our present analyses, osseointegration was confirmed at the interface of the implants placed in the regenerated bone of the MCOB-3DPL4 and cytrans groups, suggesting that the regenerated bone formed using a MCOB-3DPL4 construct is suitable for implant therapy. Over the past few years, biodegradable scaffolds have become increasingly used in the BTE applications including for the treatment of large bone defects. Bone regeneration was not observed in the pig model was mainly due to using HAOB as a xenogeneic cell source within the 3DPLA scaffold. PLA is one the most popular biomaterials that have been approved by the Food and Drug Administration (FDA) for human usage and is extensively being used in tissue engineering applications and has great potential for its use in human translational research provided the appropriate cell type is utilized. The results of our preliminary study demonstrated that the use of autologous osteoblasts combined with PLA scaffold could provide great benefits from a translational clinical perspective.

In summary, a combination of immature osteoblast–like cells and a 3DPL4 scaffold has a potential for bone tissue engineering applications that can correct large bone defects, such as those that can form in a jawbone, and may be used in the future to recover masticatory function in patients affected by such defects. Following our current observations in the mouse model, a nonclinical proof of concept will still be needed. Future evaluations of large-scale transplant material of around 2 cm^3^ to treat large bone defects will be necessary to establish effective evaluation criteria for this approach. A horizontal bone defect in a large animal model such as a micromini pigs and the autologous transplantation of porcine alveolar bone–derived immature osteoblast–like cells (PAOB)-3DPL constructs should be conducted. This will also require functional analysis, including mechanical strength testing, bone morphology measurements, and implant placement testing prior to possible human clinical trials.

## Supplementary Material

pgac151_Supplemental_Files

## Data Availability

All study data are included in the article and/Supplementary information.
