# Supplementary material for: Correction of large jawbone defect in the mouse using immature osteoblast–like cells and a 3D polylactic acid scaffold
Source: PNAS Nexus. 2022 Aug 18;1(4):pgac151. doi: 10.1093/pnasnexus/pgac151 (PMC9802318; doi:10.1093/pnasnexus/pgac151)
Supplement: pgac151_Supplemental_Files [file pgac151_supplemental_files.zip › PNASNEXUS-PNASNEXUS-2022-00126-s01.docx]

**Correction of large jaw bone defect in the mouse using immature osteoblast-like cells and a three dimensional polylactic acid scaffold**

Shigeto Suzuki ^1*^, Venkata Suresh Venkataiah ^1*^, Yoshio Yahata ^1^, Akira Kitagawa ^1,2^, Masahiko Inagaki ^3^, Mary M. Njuguna^1^, Risako Nozawa^1^, Yusuke Kakiuchi ^1^, Masato Nakano ^1^, Keisuke Handa ^1,4^ Masahiro Yamada^5^, Hiroshi Egusa^5^ and Masahiro Saito^1,2^

1.Division of Operative Dentistry, Department of Ecological Dentistry, Graduate School of Dentistry, Tohoku University, Sendai, Miyagi 980-8575, Japan
2.OsteRenatos Ltd. Sendai Capital Tower 2F, 4-10-3 Central, Aoba-ku, Sendai, Miyagi, 980-0021, Japan
3.National Institute of Advanced Industrial Science and Technology, 2266-98 Anagahora, Nagoya, Aichi 463-8560, Japan
4.Department of Oral Science, Division of Oral Biochemistry, Graduate School of Dentistry, Kanagawa Dental University, Yokosuka, Kanagawa 238-8580, Japan.

5.Division of Molecular and Regenerative Prosthodontics, Tohoku University, Graduate School of Dentistry, Sendai, Miyagi 980-8575, Japan

Corresponding author: Masahiro Saito DDS. Ph.D.

**Email:** masahiro.saito.c5@tohoku.ac.jp

**Author Contributions: * S.S and * V.S.V contributed equally to this study.** Conceptualization, V.S.V., M.S., and K.H.; original draft preparation, V.S.V., Y.Y., A.K., and M.I.; review and editing, V.S.V., M.N., Y.K., and S.S. All authors have read and agreed to the published version of the manuscript.

**Competing Interest Statement:** The authors declare no conflicts of interest.

**Classification:** Biological Sciences Medical Sciences

**Keywords:** Human alveolar osteoblasts, mice calvaria osteoblasts, bone regeneration, polylactic acid scaffold, functional bone.

**This PDF file includes:**

Abstract

Significance statement

Main Text

Figure Legends

**Abstract.**Bone tissue engineering has been developed using a combination of mesenchymal stem cells (MSCs) and calcium phosphate-based scaffolds. However, these complexes cannot regenerate large jawbone defects. To overcome this limitation of MSCs and ceramic scaffolds, a novel bone regeneration technology must be developed using cells possessing high bone forming ability and a scaffold that provides space for vertical bone augmentation. To approach this problem in our study, we developed alveolar bone-derived immature osteoblast-like cells (HAOBs), which have the bone regenerative capacity to correct a large bone defect when used as a grafting material in combination with polylactic acid fibers that organize the three-dimensional structure and increase the strength of the scaffold material (3DPL). HAOB-3DPL constructs could not regenerate bone via xenogeneic transplantation in a micromini pig alveolar bone defect model. However, the autogenic transplantation of mice calvaria-derived immature osteoblast like cells (MCOBs) isolated using the identical protocol for HAOBs and mixed with 3DPL scaffolds successfully regenerated the bone in a large jawbone defect mouse model, compared to the 3DPL scaffold alone. Nanoindentation analysis indicated that the regenerated bone had a similar micromechanical strength to native bone. In addition, this MCOB-3DPL regenerated bone possesses osseointegration ability wherein a direct structural connection is established with the titanium implant surface. Hence, a complex formed between a 3DPL scaffold and immature osteoblast-like cells such as MCOBs represents a novel bone tissue engineering approach that enables the formation of vertical bone with the micromechanical properties required to treat large bone defects.

**Significance Statement**

Large bone defects that cannot heal spontaneously are increasing in association with an aging society in the inability to adequately treat these defects represents an unmet medical need. This study shows that a complex of osteogenic cells, such as immature osteoblast like cells, and a poly-lactic acid polymer based on three-dimensional biodegradable scaffolds has the potential to overcome this drawback. These complexes were found to successfully induce bone regeneration in a vertical direction upon autologous transplantation into mouse jawbone defects and possess the micromechanical properties as native bone and suitable for prosthetic rehabilitation, such as dental implantation. These complexes of immature osteoblasts and three-dimensional scaffolding represent a novel bone regeneration material for the treatment of large alveolar bone defects.

**Introduction**

With recent advances in bone tissue engineering technology, the availability of bone substitute materials such as carbonate apatite, demineralized bovine bone, beta-tricalcium phosphate (β-TCP), and hydroxyapatite have reduced the burden on patients with regard to autologous transplantation, which requires additional surgical invasion for bone collection. Notably however, most of these materials are granular type compounds that are only effective in treating mild bone defects surrounded by existing native bone(1–5). Three-dimensional bone substitute materials combined with a fibrillar matrix such as collagen or polymers have thus been developed for more complicated defects. Materials of this type containing a porous body, mainly comprising collagen and octacalcium phosphate, have been shown to be effective for bone forming treatments prior to implant placement, including maxillary sinus floor fistula surgery and tooth extraction socket preservation therapy, but have not been effective for large jaw bone defects (6, 7).

Regeneration therapies for large bone defects will require the capacity to rapidly form bone in the vertical dimension. It has been shown previously that this type of growth can be achieved through a three-dimensional arrangement of osteogenic cells, such as immature osteoblasts, in a scaffold. Human alveolar bone derived immature osteoblasts (HAOBs) show higher osteogenic ability than MSCs, can be expanded from the alveolar bone of patients older than 60 years, and can produce bone matrix *in vivo* without the need for treatment with osteogenic induction medium (8). HAOBs can be also obtained from alveolar bone during dental surgery by collagenase digestion (9, 10), differentiated into osteoblasts *in vitro* by exposure to osteogenic differentiation medium, and induce bone formation upon subcutaneous implantation into immunodeficient mice (8). A principal advantage of bone forming cells such as HAOBs is that they can reduce the surgical burden on patients undergoing autologous bone graft transplantation, which is the current standard treatment for large bone defects.

Horizontal bone defects are large jaw bone defects that do not heal spontaneously because they require vertical bone augmentation, and this cannot be achieved efficiently with existing bone regeneration therapies (11). The number of patients who cannot receive dental implants due to large jaw bone defects has increased dramatically in the past two decades and is expected to further expand in the future. The current standard treatment for these anomalies is autologous bone transplantation which requires the collection of bone from the jaw or iliac crest, and its subsequent transplantation to bone defect site for more than six months to generate sufficient replacement bone, followed by implant therapy (12, 13). HAOBs are a candidate cell source for the treatment of large alveolar bone defects that require vertical bone augmentation. One of the critical requirements for successful regeneration of large bone defects is the development of a scaffold suitable for promoting osteoblast differentiation. Hence, the development of materials with appropriate stiffness that can promote osteogenic differentiation of HAOBs and withstand the mechanical load at transplantation sites is essential for this purpose (14).

In our present study, we describe the development of a three-dimensional cotton-like scaffold structure produced by novel electrospinning technology using poly-L-lactic acid and gelatin (3DPL). This complex is suitable for HAOB differentiation to osteoblasts to regenerate large jawbone defects. We initially found that the xenotransplantation of HAOB-3DPL constructs, preconditioned in osteoblastic differentiation medium, did not promote bone formation in a micro mini pig jawbone defect model. To resolve this problem, we here established a mouse alveolar bone defect model and investigated the bone regenerating ability of an autogenic transplantation of mice calvaria-derived osteoblasts (MCOBs), isolated using a protocol for HAOB preparations, and combined these cells with a 3DPL scaffold. We found using bone morphometric analysis that these MCOB-3DPL constructs regenerated new bone with suitable micromechanical properties for implant therapy. Our present study findings suggest that the autogenic transplantation of immature osteoblast-like cells and a 3DPL nanoarchitecture scaffold represents a viable new bone tissue engineering methodology for large jawbone defects.

**Results**

**Design, fabrication, and characterization of 3-dimensional polylactic acid scaffolds**

To achieve vertical bone augmentation, a 3-dimensional (3D) polylactic acid scaffold (3DPL) was fabricated with high mechanical properties and a controlled scaffold microstructure to tailor the pore size and distribution, and the biodegradability rate, as illustrated in Figures 1*A-C.* Adjustable 3DPL scaffolding was generated via electrospinning method using polylactic acid and gelatin. The fibers formed by electrospinning are similar to the collagen fibers of the extracellular matrix of bone and can be utilized as a porous base material for cell attachment and proliferation. In our present study, electrospinning is performed using polylactic acid and gelatin as raw materials (Fig. 1*A*).

PLLA/gelatin cotton-like fabrics (hereafter referred as PLA fabrics) were then formed by collection with a cylindrical target (Fig. 1*B*), compression processing of stacked PLA fabrics (8 x 8 cm^2^) was performed (Fig. 1*C*), and the size (length 5 mm x width 3 mm x height 2 mm) was adjusted by laser cutting to create the 3DPL scaffold material (Fig. 1*D*). The thickness of the 3DPL scaffold material was confirmed by vernier caliper (Fig. S1). The fiber diameter distribution of all the different lots of PLLA/gelatin floccular fibers was estimated from the Laser confocal microscope (LCM) using the accompanying LCM analysis software (LEXT OLS application program, OLYMPUS, Japan) (Fig.S2). The x-axis represents the diameter of electrospun fibers, while the Y-axis contains the diameter distributions obtained by analyzing 180 fibers in each specimen. The average fibre diameter lies in the range of 1.2μm. No differences in fiber diameter distribution were found in the beeswarm plots of the eight lots of PLL/gelatin floccular fibers (Fig. S2). Four kinds of 3DPL scaffolds (3DPL1, -2, -4, and -6) with varying degrees of mechanical stiffness were prepared by compression molding. Here, the number of 3DPL scaffold is the number of stacked PLA fabrics in compression process (for example: 3DPL4 when the number of stacked PLA fabrics is 4). Hence, the density of the scaffold fibers increases with higher stacked PLA fabrics rendering to increased stiffness, but the fiber diameter remains constant for all types of 3DPL scaffolds. The topology of the 3DPL1 (Fig. 1*E*), 3DPL2 (Fig. 1*F*), 3DPL 4 (Fig. 1*G*), and 3DPL 6 (Fig. 1*H*) scaffold surfaces was demonstrated by SEM analysis which revealed a characteristic 3D morphology with randomly oriented polylactic fibers. By contrast, the 2-dimensional PL scaffold (2DPL), which has a non-woven fabric structure, showed a linear fiber structure (Figure.1*I*). The diameter of the fibers used for the 3DPL scaffolds was set to 1.5 μm to meet the requirements for bone tissue regeneration (Fig. 1*J*) and was confirmed by laser confocal microscopy (LCM). The chemical identity of the 3DPL scaffold was analyzed using Fourier-transform infrared spectroscopy (FTIR). The FTIR spectrum in Fig 1 *K*(iii) showed characteristic absorption bands at 1750 cm-1 related to C=O (ester stretch) and a short bands at 1180 and 1080 cm-1 (ester bend), which represents the backbone ester group of PLLA. Fig 1 *K*(iv), in addition to the hydrogen bond and amide as a peak at 3500 and 3300 cm-1, the amide I and II bands are seen as the peaks at 1640 cm-1 and 1550 cm-1 are related to gelatin. These spectra indicated that the tested samples were typically made of PLLA/gelatin without the presence of significant contaminants (Fig. 1*K*). As explained above, four types of 3DPL scaffolds were prepared to assess their suitability for immature osteoblast attachment, proliferation, and differentiation. 3DPL1 and -6 showed the lowest and highest stiffness, respectively. The mechanical testing results for the panel of 3DPL scaffolds are shown in Figure 1*L*. A significant increase in stiffness was observed as the fiber density increased in the 3DPL2, -4, and -6 scaffolds, respectively. These scaffolds were kept at 37°C under wet conditions for 1, 3, and 5 months, and the mechanical properties were deteriorated due to the hydrolysis of PLA. The mechanical strength was found to be maintained to the extent that it could be handled or shaped (Fig. 1*M)*. In a compression test on a 1 cm^3^ test piece with a fiber density equivalent to that of 3DPL scaffolds, the elastic restoration of the scaffold shape was observed during by unloading even after a 90% compressive deformation, and the samples were not crushed. In that experiment, the restoration rate for the compression variant was about 80% (Fig. 1N).These overall structural parameters of the 3DPL scaffold enabled its use in cell seeding experiments for bone tissue engineering.

**Xenotransplantation of the HAOB-3DPL4 complex does not induce bone regeneration in the pig**

The standard approach for bone tissue engineering involves a combination of osteogenic cells and a biocompatible scaffold material. We seeded a HAOB cell suspension onto our 3DPL4 scaffold to prepare a tissue-engineered construct and investigated its osteogenic characteristics *in vitro* and bone regeneration ability *in vivo* via transplantation into a pig alveolar bone defect model.

We first conducted *in vitro* osteogenic gene expression analysis to identify the most suitable osteogenic stimulant for the HAOB-3DPL4 constructs. We developed a 28-day culture model that included an initial proliferation phase of 7 days in basal medium to allow HAOBs to proliferate and migrate within the scaffold, followed by a 7- and 14-day induction with osteogenic stimulants (Fig. 2*A*). The results of Q-PCR analysis provided evidence of osteogenic differentiation by detecting the expression of OSTERIX, RUNX2, TYPE I COLLAGEN, and BONE SIALOPROTEIN (BSP) at both 14 (Fig. 2*B*) and 21 (Fig. 2*C*) days. No significant difference was observed in osteogenic gene expression by HAOB treated with SAG, TH, SAG+TH and BMP2 groups at 14 days of incubation. However, TYPE I COLLAGEN was significantly decreased by HAOB treatment with BMP2 and SAG compared to TH and SAG+TH at 21 days.

We next investigated the *in vivo* bone regenerative ability of HAOB-3DPL4 constructs following their transplantation into a micromini pig alveolar bone defect model. Defects filled with scaffold alone served as a control group. Healing progressed uneventfully without an intense inflammatory reaction during the 8-week observation period. The animals were sacrificed at 8 weeks post-transplantation and the jaws were collected to investigate new bone formation by μCT and histological analysis. 3D reconstructions of the μCT images revealed new alveolar bone formation (Fig. 2D, yellow region) in both the HAOB-3DPL4 and the 3DPL4 (control) groups. Quantitative bone volume analysis of the defect area indicated no significant differences in the bone volume, trabecular volume, and bone mineral density between the control and HAOB-3DPL4 groups (Fig. 2*E*). We next performed histological analysis to evaluate new bone regeneration. The experimental area was sectioned and stained with hematoxylin-eosin (HE) and Masson's Trichrome (MT). The resulting HE and MT-stained photomicrographs indicated minimal bone formation above the base of the defect (outlined by dotted line) in both the control and HAOB-3DPL4 groups (Fig. 2*F*). A possible reason the lack of any significant new bone regeneration in the HAOB-3DPL4 group could be a rejection of the HAOB cells by the mini pig host immune system.

**The autologous transplantation of MCOB-3DPL4 constructs successfully induces bone regeneration in a mouse alveolar bone defect model**

We next investigated the bone regenerative ability of mouse calvaria-derived immature osteoblast like cells (MCOBs) that had been collected in the same way as HAOBs and combined with the 3DPL4 scaffolds (Fig. S6). These constructs were then transplanted into large alveolar bone defects in a mouse model. We first investigated the *in vitro* osteogenic ability of the MCOBs when cultured in osteogenic differentiation medium (ODM) at 10 days incubation. Under this ODM treatment, these cells exhibited strong ALP activity, as indicated by positive violet staining, and intense staining of calcified nodules by alizarin red (Fig.3*A*, lower panel). MCOBs without ODM induction displayed weak ALP and alizarin staining, indicating their undifferentiated state (Fig. 3*A, Upper panel*). Quantitative PCR analysis revealed that osteogenic genes including osterix, osteocalcin, and runx2 were more highly expressed in the MCOBs grown in ODM, further indicating their osteogenic differentiation ability (Fig. 3*B*). The adhesion of the required bone cells to a scaffold is an important prerequisite for successful bone tissue engineering. Following confirmation of the osteogenic ability of MCOBs, we investigated their attachment capacity to the 3DPL4 scaffold by SEM analysis two hours after seeding (Fig. 3*C,* left*)*. High magnification analysis of these constructs showed that the majority of the attached cells were round in shape with cell-cell and cell-3DPL4 contact, indicated by red arrows and that polylactic fibers were barely visible (Fig. 3*C* right). SEM analysis of the 3DPL4 scaffold without cells (Fig. 3*D,* left) clearly showed the presence of only PL fibers at high magnification (Fig. 3*D,* right). To then investigate the *in vivo* bone forming ability of the MCOB-3DPL4 constructs, we performed subcutaneous implantation into C57BL / 6N mice as a model of autologous transplantation. HE staining of transplanted scaffold constructs seeded with MCOBs at both 4- and 8-weeks post-transplantation indicated that bone-like tissue formation had occurred within the 3DPL4 fibers (Fig. 3*E,* upper and lower panel). In contrast, the 3DPL4 scaffolds without cells showed only connective tissue formation (Fig. 3*F,* upper and lower panel). These data confirmed that MCOB-3DPL4 scaffold constructs are osteoinductive and that MCOBs seeded within the scaffold contribute to new bone tissue formation. To further verify the jawbone regeneration ability of the MCOB-3DPL4 constructs, we investigated whether they promoted the regeneration of functional bone in a maxillary bone defect mouse model (Fig. S6, Movie S1). 2D μCT images of the MCOB-3DPL4 transplant group confirmed bone regeneration in the defect area, as indicated by increased radiodensity at both 4- and 8- weeks post transplantation, reaching up to the cemento enamel junction (CEJ) (Fig. 4*A*, Fig. S8*A* and *B*). A control group in which the defect was filled with cytrans, a calcium carbonate-based bone substitutes approved for clinical applications, showed a comparable level of new bone formation. However, the increased radiodensity in the cytrans group was partially due to retained carbonate apatite granules. In the 3DPL4 and empty defect comparison groups, a minimal amount of bone formation was observed. Quantification of the new bone formation in the defect area indicated no significant difference between any of the experimental groups at 4 weeks (Fig. 4*B*, left panel), but a significantly higher level in the MCOB-3DPL4 and cytrans groups compared to the empty and 3DPL4 groups at 8 weeks post transplantation (Fig. 4*B* right panel), and no significant difference between the MCOB-3DPL4 and cytrans groups at this timepoint. From the 3D reconstructed μCT images, buccal bone defects were clearly observed in the 3DPL4 and empty defect animals but completely recovered in the MCOB-3DPL4 and cytrans groups. Notably however, scattered, and residual carbonated apatite granules were observed in the cytrans group (Fig. 4*C* and Fig. S8*B*).

**Histological analysis of the regenerated bone induced by MCOB-3DPLA**

**constructs**

To histologically investigate the regeneration of bone induced by MCOB-3DPLA, HE staining was performed within the bone defect area in the mouse model. M1 (maxillary first molar) area in the all the histological images represents the bone defect area. (Fig 4*D* and Fig S9). In the MCOB-3DPL4 transplant group nearly half of the defect area was filled with new bone at 4 weeks (Fig. S9) and mature, organized bone tissue along with bone marrow was formed at 8 weeks (Fig. 4*D*) post-transplantation. At higher magnification observations in the MCOB-3DPL4 group, new bone formation was found to be incorporated around 3DPL fibers (arrowheads) at 8 weeks post-transplantation (Fig. 4*D*, lower row). In the cytrans control group, partial new bone formation was observable around the residual carbonate apatite granules within the defect area at both 4- and 8-weeks post-transplantation (Fig. 4*D*, Fig. S9). The 3DPL4 control group showed the presence of inflammatory infiltrates in the defect area, while the empty group showed only partial granulation tissue and bone formation in the center of the defect area, at 4- and 8-weeks post-transplantation. (Fig. 4*D*, Fig. S9). These histological findings were thus consistent with the μCT data, both of which demonstrated a high amount of bone formation in the MCOB-3DPL4 and cytrans groups.

Overall, these data indicate that the defect areas transplanted with MCOB-3DPL4 and cytrans material showed active remodeling processes in the newly regenerated bone.

**Biomechanical properties of the newly regenerated bone**

We next investigated the micromechanical properties of the regenerated bone in the mouse maxilla defect model using nanoindentation measurements, an effective technique for assessing the hardness of bone tissue (Fig. S11).

The micromechanical properties of the regenerated bone from the cytrans granules showed a greater ability to resist elastic deformation (Fig. 5*A* and *B*) due to retained residual carbonate apatite granules. The highest tissue hardness in the regenerated bone was observed in the cytrans group, followed by the 3DPL4 (represents native bone), MCOB-3DPL4 and empty defect groups. However, the hardness of the newly formed bone tissue was not significantly different between the experimental groups (Fig. 5*A*). The elastic modulus was also higher in the regenerated bone of the cytrans group compared to 3DPL4 and empty defect groups, but this difference between the cytrans and MCOB-3DPL4 groups was also not significant (Fig. 5*B*). However, elastic modulus of MCOB-3DPL4 group is comparable to 3DPL4 (represents native bone) and empty group. Overall, these data indicated that the regenerated bone in the MCOB-3DPL4 group possessed similar micromechanical properties to the native bone of the maxilla. The osseointegration ability of the regenerated bone was next evaluated by placement of a dental implant to assess the tolerance to implant therapy (Movie S2). The implants were placed in the area of regenerated bone (M1 area) in all of the experimental groups and osseointegration was tested after 4 weeks (Fig. S14) by μCT and histological analysis. The implant dropout ratio (remaining implants/implantation number) was 5/5 in both the MCOB-3DPL4 and 3DPL4 groups, which was superior to 3/5 in the control (empty) group and 4/5 in the cytrans group. 2D μCT analyses (Fig. 5*C*) and 3D reconstructions (Fig. 5*D*) revealed that there was no complete osseointegration of the implants within the regenerated bone in any of the groups. We evaluated this further by HE staining and found evidence of good osseointegration in the MCOB-3DPL4 and cytrans groups, and partial osseointegration in the 3DPL4 and empty defect groups (Fig. 5*E*).

**Discussion**

New bone tissue engineering technologies have been developed to regenerate large bone defects that can adequately recapitulate bone developmental processes(15). We demonstrate in our present study that 3DPL4, a poly-lactic acid-based nanoscale architecture scaffold, has the appropriate degree of mechanical strength to enable osteoblast differentiation and provide a suitable microenvironment for bone formation upon autologous transplantation of MCOBs into a large alveolar bone defect in the mouse maxilla. In addition, the MCOB-3DPL4 constructs were found to support the regeneration of bone in a vertical direction with sufficient mechanical strength for implant placement.
Macroscale architecture scaffolds have been developed previously for bone regeneration, mainly using calcium phosphate-based scaffolds (16–19). An essential characteristic of a macro porous calcium phosphate-based scaffold is the use of a combination of collagen and apatite to promote the attachment of cells with a flattened shape (20). In contrast to calcium phosphate-based materials, more recent nanofiber technologies have enabled the generation of nanoarchitecture scaffolds with pore sizes that are smaller than a cell diameter, which provides a three-dimensional morphology that mimics the cells within connective tissues such as bone (21–24). The mechanical properties of the materials used in scaffolds influence the ability of cells to differentiate, and scaffolds of sufficient mechanical strength are required at the transplanted site for successful regeneration therapy (25). The electrospinning technique is a technology in which a high voltage is applied to a polymer solution to stretch the fibers with the electrostatic force of an electric field in the order of micro to nanometers(26–29). In conventional electrospinning, the formed electrospun fibers are strongly aggregated on the electrode and a 2D sheet-like non-woven fabric is produced (30). However, these structures cannot provide a sufficient space for cell proliferation and are not suitable as a bone replacement material. To solve this problem, 3DPL was developed with intercommunicating pores that have characteristics of a fibrous porous body, that can withstand compression with 90% deformation, have an easily adjustable elastic modulus by fiber density, and provide an environment suitable for the osteoblast differentiation of HAOBs and MCOBs. This outcome resulted from increasing the width of the PL fibers to maintain porosity and layering to maintain their strength. This outcome was a result of formation of cotton-like PLA fibers with appropriate fiber diameter using emulsion-electrospinning. Such cotton-like PLA fibers have an enough flexibility and a sufficient modulus of elasticity by themselves to hold the spaces among the fibers. As seen in the compression test of the 3DPL4 scaffold, such flexibility and resiliency of the cotton-like PLA fiber was maintained even after compression process for fiber density adjustment.

Our present results indicated that HAOBs synthesize an extracellular matrix on a 3DPL4 scaffold via osteoblast differentiation, but the subsequent xenotransplantation of these constructs into a pig bone defect model did not induce bone formation. This indicated that HAOBs differ from MSCs and cannot be xeno- or allo-transplanted due to a low immunological tolerance of the host animal. We thus conducted autogenic transplantation using MCOBs in a mouse maxillary bone defect model. Vertical bone regeneration was achieved in this system by engrafting MCOB-seeded 3DPL4 constructs without preconditioning for osteoblast differentiation. In the current regenerative medicine technology field, the transplantation of undifferentiated cells has been found to regenerate body tissue functions in certain settings. For example, undifferentiated cardiomyocytes derived from induced pluripotent stem cells (iPSCs) were reported to differentiate into cardiomyocytes that restored cardiac function following transplantation into a rat heart failure model (31). In addition, the transplantation of undifferentiated Langerhans islet β cells, derived from human embryonic stem cells (ESCs), into diabetic immunodeficient mice was found to successfully recover the blood glucose levels in a treatment model of type I diabetes(32). In the present study we found that xenogeneic transplantation of HAOB-3DPL4 scaffold constructs within the minipig alveolar bone defect generated poor bone formation. No significant differences were observed compared to the 3DPL4 scaffold without HAOB. However, MCOB, which was isolated similar to that of HAOB, combined with the 3DPL4 scaffold generated new bone formation and was significantly higher than the 3DPL4 scaffold without MCOB. This kind of anomalous response between two animal models was mainly due to the type of transplantation, xenogeneic and autologous. Interestingly many studies show similar results regarding the efficacy of xenogeneic transplants. For example, although MSCs are anticipated to have Immunosuppressive and immunomodulatory functions, they have shown conflicting results when used for bone regeneration therapeutics following xenogeneic transplantation across species (33–36). Our study demonstrated that xenogeneic transplantation of HAOB into the Pig bone defect model showed no new bone formation due to undesired immune response mounted by pig host immune cells and possibly leading to transplanted cells' death. However, autologous transplantation of MCOB into the mouse defect model showed significantly higher bone formation due to the absence of immune response against transplanted cells, secretion, and mineralization of collagen matrix. The results of our study demonstrate that reduced new bone formation occurred in the xenogeneic transplant groups from micro-CT and histological findings, despite the absence of evidence of immune rejection. Our study assumed that rejection of HAOB contributed causally to the results found in our study. The current study was designed to focus specifically on the efficacy of cell-scaffold constructs in new bone regeneration and investigate the mechanical properties of the regenerated bone. However, survival of the transplanted cells is a major challenge and one of the important criteria determining the success or failure of treatment. Since autologous transplantation was performed in this study, MCOB did not reject immediately upon transplantation, but we do not know how long cells survived at the transplantation site. To address this problem, MCOB isolated from GFP-transgenic mice will be utilized for tracing cells following transplantation in our future study. Nevertheless, the results of the present study support the evidence that autologous transplantation of osteoblasts is superior in promoting new bone formation. Thus, this autologous approach should be supported and considered for future translational research in bone regeneration therapeutics.

PL scaffolds are biocompatible and therefore have a potentially broad range of applications in the medical and dental field, including bone tissue engineering. In a previous study however, the transplantation of PL scaffold alone was reported to induce inflammation (37) which was suppressed by silver ions (38). Similarly, in this present study a 3DPL scaffold alone induced inflammation when transplanted into our mouse model of maxillary bone defects. We therefore speculated that the addition of MCOBs may also suppress the 3DPL-induced inflammatory response during their differentiation into osteoblasts on this scaffold. From our results and previous findings, it will clearly be necessary to prevent 3DPL-induced inflammation in a real-world clinical setting.

The challenge for regenerative therapy in correcting large bone defects requires functional analysis to verify whether regenerated bone formed by tissue engineering can actually be used in a clinical application (39). In addition, mechanical properties of tissue engineering scaffolds are vital to ensure the long-term structural and functional viability of cells in both in-vitro and in-vivo (40). Human cells sense the mechanical properties of the extracellular matrix, making them responsive to the mechanical cues from the environment (41–43). Our invitro studies showed increased staining intensity of alizarin in scaffolds with higher stiffness (3DPL4 and 3DPL6) than with low stiffness (3DPL1, 3DPL2 and 3DPL3 ), indicating that stiffer scaffolds are more appropriate for cell adhesion, differentiation, secretion of extracellular matrix and mineralization. This observation goes along with other studies which describes the stiffness of the scaffold plays an essential role in cell adhesion and differentiation and is better suitable for bone regeneration applications. From a bone tissue regeneration perspective, scaffolds with high stiffness favour osteogenic differentiation of MSCs (40, 42). We therefore used 3DPL4 scaffold for all further experiments. To determine whether the regenerated bone formed by MCOB-3DPL4 constructs induced functional bone formation that could withstand masticatory function, the regenerated bone was analyzed using a nanoindentation test that can measure bone strength and elastic modulus on a small scale, enabling the quantification of bone mechanical properties (44). The calcification levels affect the strength and elasticity of bone, and are indicative of bone maturation and an increased mineral content, indicating that mechanical strength is a regulatory factor in the bone remodeling processes that play an important role in bone formation and homeostasis (45, 46). A carbonate apatite material such as cytrans has been reported to activate bone metabolism and bone regeneration at an early stage, and clinical studies using this scaffold have indicated that implant therapy can be performed with the regenerated bone induced by maxillary sinus floor elevation (47). However, there are no reported studies on the functional evaluation, including the micromechanical strength of the regenerated bone, induced by the same material. Our current study findings indicated that the regenerated bone in the cytrans transplanted group had higher micromechanical strength than the native maxillary bone, suggesting that the bone micromechanical properties had been affected by remaining cytrans within the maxillary bone defect area. On the other hand, the regenerated bone formed in the MCOB-3DPL4 transplanted group had identical micromechanical strength to the bones within the control group and also the 3DPLA alone transplanted group, indicating that the regenerated bone induced by MCOB-3DPL4 had the same micromechanical property as native bone, and that the PL fibers which had been incorporated into the regenerated bone did not affect bone strength. Osseointegration refers to bone fusion at the interface between dental implants and bone (48, 49), and has been observed in regenerated bone induced by cytrans. In our present analyses, osseointegration was confirmed at the interface of the implants placed in the regenerated bone of the MCOB-3DPL4 and cytrans groups, suggesting that the regenerated bone formed using a MCOB-3DPL4 construct is suitable for implant therapy. Over the past few years, biodegradable scaffolds have become increasingly used in the BTE applications including for the treatment of large bone defects. Bone regeneration was not observed in the pig model was mainly due to using HAOB as a xenogeneic cell source within the 3DPLA scaffold. PLA is one the most popular biomaterial that has been approved by the Food and Drug Administration (FDA) for human usage and is extensively being used in tissue engineering applications and has great potential for its use in human translational research provided the appropriate cell type is utilized. The results of our preliminary study demonstrated that the use of autologous osteoblasts combined with PLA scaffold could provide great benefits from a translational clinical perspective.

In summary, a combination of immature osteoblast-like cells and a 3DPL4 scaffold has a potential for bone tissue engineering applications that can correct large bone defects, such as those that can form in a jawbone, and may be used in the future to recover masticatory function in patients affected by such defects. Following our current observations in the mouse model, a non-clinical proof of concept will still be needed. Future evaluations of large-scale transplant material of around 2 cm^3^ to treat large bone defects will be necessary to establish effective evaluation criteria for this approach. A horizontal bone defect in a large animal model such as a micromini pigs, and the autologous transplantation of porcine alveolar bone derived immature osteoblast-like cells (PAOB)-3DPL constructs should be conducted. This will also require functional analysis including mechanical strength testing, bone morphology measurements and implant placement testing prior to possible human clinical trials.

**Data Availability:** All study data are included in the article and/Supplementary information.

**Acknowledgments**

We greatly thank Dr. Shunsuke Ohba, Dr. Satoru Yamada, Dr Kenji Hara Dr. Masafumi Kanehira and Dr. Makoto Aino for beneficial advice and discussion, and Ms. Yayoi Aoyama for conducting the immunohistological work.

This work was supported by Japan Society for the Promotion of Science (JSPS) KAKENHI, Grant-in-Aid for Scientific Research(B) 21H03116 and Early-Career Scientists 21K16962.

**References**

1. K. Hayashi, R. Kishida, A. Tsuchiya, K. Ishikawa, Granular Honeycombs Composed of Carbonate Apatite, Hydroxyapatite, and β-Tricalcium Phosphate as Bone Graft Substitutes: Effects of Composition on Bone Formation and Maturation. *ACS Appl. Bio Mater.* **3**, 1787–1795 (2020).

2. N. Sato, *et al.*, Comparison of the vertical bone defect healing abilities of carbonate apatite, β-tricalcium phosphate, hydroxyapatite and bovine-derived heterogeneous bone. *Dent. Mater. J.* **39**, 309–318 (2020).

3. S. Sotome, *et al.*, Efficacy and safety of porous hydroxyapatite/type 1 collagen composite implantation for bone regeneration: A randomized controlled study. *Journal of Orthopaedic Science* **21**, 373–380 (2016).

4. T. Funayama, H. Noguchi, T. Tsukanishi, M. Sakane, Histological Analysis of Bone Bonding and Ingrowth into Connected Porous Hydroxyapatite Spacers in Spinal Surgery. *KEM* **529–530**, 309–312 (2012).

5. T.-Y. Wang, S.-L. Xu, Z.-P. Wang, J.-Y. Guo, Mega-oss and Mega-TCP versus Bio-Oss granules fixed by alginate gel for bone regeneration. *BDJ Open* **6**, 14 (2020).

6. K. Miura, *et al.*, First clinical application of octacalcium phosphate collagen composite on bone regeneration in maxillary sinus floor augmentation: A prospective, single‐arm, open‐label clinical trial. *J. Biomed. Mater. Res.* **108**, 243–252 (2020).

7. T. Kawai, *et al.*, Clinical study of octacalcium phosphate and collagen composite in oral and maxillofacial surgery. *J Tissue Eng* **11**, 204173141989644 (2020).

8. M. Aino, *et al.*, Isolation and characterization of the human immature osteoblast culture system from the alveolar bones of aged donors for bone regeneration therapy. *Expert Opinion on Biological Therapy* **14**, 1731–1744 (2014).

9. A. Pilloni, *et al.*, Analysis of human alveolar osteoblast behavior on a nano-hydroxyapatite substrate: an in vitro study. *BMC Oral Health* **14**, 22 (2014).

10. D. Marolt, *et al.*, Engineering bone tissue from human embryonic stem cells. *Proceedings of the National Academy of Sciences* **109**, 8705–8709 (2012).

11. Y. Ding, *et al.*, Horizontal bone augmentation and simultaneous implant placement using xenogeneic bone rings technique: a retrospective clinical study. *Sci Rep* **11**, 4947 (2021).

12. A. Sakkas, F. Wilde, M. Heufelder, K. Winter, A. Schramm, Autogenous bone grafts in oral implantology—is it still a “gold standard”? A consecutive review of 279 patients with 456 clinical procedures. *Int J Implant Dent* **3**, 23 (2017).

13. W. L. Grayson, *et al.*, Stromal cells and stem cells in clinical bone regeneration. *Nat Rev Endocrinol* **11**, 140–150 (2015).

14. S. Prasadh, R. C. W. Wong, Unraveling the mechanical strength of biomaterials used as a bone scaffold in oral and maxillofacial defects. *Oral Science International* **15**, 48–55 (2018).

15. E. J. Sheehy, D. J. Kelly, F. J. O’Brien, Biomaterial-based endochondral bone regeneration: a shift from traditional tissue engineering paradigms to developmentally inspired strategies. *Materials Today Bio* **3**, 100009 (2019).

16. K.-S. Lew, R. Othman, K. Ishikawa, F.-Y. Yeoh, Macroporous bioceramics: A remarkable material for bone regeneration. *J Biomater Appl* **27**, 345–358 (2012).

17. J. Vivanco, A. Aiyangar, A. Araneda, H.-L. Ploeg, Mechanical characterization of injection-molded macro porous bioceramic bone scaffolds. *Journal of the Mechanical Behavior of Biomedical Materials* **9**, 137–152 (2012).

18. R. Wu, *et al.*, Bone tissue regeneration: The role of finely tuned pore architecture of bioactive scaffolds before clinical translation. *Bioactive Materials* **6**, 1242–1254 (2021).

19. M. Sari, P. Hening, Chotimah, I. D. Ana, Y. Yusuf, Bioceramic hydroxyapatite-based scaffold with a porous structure using honeycomb as a natural polymeric Porogen for bone tissue engineering. *Biomater Res* **25**, 2 (2021).

20. Q. L. Loh, C. Choong, Three-Dimensional Scaffolds for Tissue Engineering Applications: Role of Porosity and Pore Size. *Tissue Engineering Part B: Reviews* **19**, 485–502 (2013).

21. J. Rnjak-Kovacina, A. S. Weiss, Increasing the Pore Size of Electrospun Scaffolds. *Tissue Engineering Part B: Reviews* **17**, 365–372 (2011).

22. N. Abbasi, S. Hamlet, R. M. Love, N.-T. Nguyen, Porous scaffolds for bone regeneration. *Journal of Science: Advanced Materials and Devices* **5**, 1–9 (2020).

23. M. M. Stevens, J. H. George, Exploring and Engineering the Cell Surface Interface. *Science* **310**, 1135–1138 (2005).

24. L. Zhu, D. Luo, Y. Liu, Effect of the nano/microscale structure of biomaterial scaffolds on bone regeneration. *Int J Oral Sci* **12**, 6 (2020).

25. X. Yu, X. Tang, S. V. Gohil, C. T. Laurencin, Biomaterials for bone regenerative engineering. *Advanced healthcare materials* **4**, 1268–1285 (2015).

26. K. Maji, K. Pramanik, Electrospun scaffold for bone regeneration. *International Journal of Polymeric Materials and Polymeric Biomaterials*, 1–16 (2021).

27. U. Stachewicz, *et al.*, 3D imaging of cell interactions with electrospun PLGA nanofiber membranes for bone regeneration. *Acta Biomaterialia* **27**, 88–100 (2015).

28. R. Fang, E. Zhang, L. Xu, S. Wei, Electrospun PCL/PLA/HA Based Nanofibers as Scaffold for Osteoblast-Like Cells. *J. Nanosci. Nanotech.* **10**, 7747–7751 (2010).

29. S.-H. Shin, O. Purevdorj, O. Castano, J. A. Planell, H.-W. Kim, A short review: Recent advances in electrospinning for bone tissue regeneration. *J Tissue Eng* **3**, 204173141244353 (2012).

30. G. Poologasundarampillai, *et al.*, Cotton-wool-like bioactive glasses for bone regeneration. *Acta Biomaterialia* **10**, 3733–3746 (2014).

31. E. Karbassi, *et al.*, Cardiomyocyte maturation: advances in knowledge and implications for regenerative medicine. *Nat Rev Cardiol* **17**, 341–359 (2020).

32. A. Rezania, *et al.*, Maturation of Human Embryonic Stem Cell–Derived Pancreatic Progenitors Into Functional Islets Capable of Treating Pre-existing Diabetes in Mice. *Diabetes* **61**, 2016–2029 (2012).

33. P. Niemeyer, *et al.*, Xenogenic Transplantation of Human Mesenchymal Stem Cells in a Critical Size Defect of the Sheep Tibia for Bone Regeneration. *Tissue Engineering Part A* **16**, 33–43 (2010).

34. A. Rapp, *et al.*, Autologous Mesenchymal Stroma Cells Are Superior to Allogeneic Ones in Bone Defect Regeneration. *IJMS* **19**, 2526 (2018).

35. A. Longoni, *et al.*, Endochondral Bone Regeneration by Non-autologous Mesenchymal Stem Cells. *Front. Bioeng. Biotechnol.* **8**, 651 (2020).

36. P. Niemeyer, K. Szalay, R. Luginbühl, N. P. Südkamp, P. Kasten, Transplantation of human mesenchymal stem cells in a non-autogenous setting for bone regeneration in a rabbit critical-size defect model. *Acta Biomaterialia* **6**, 900–908 (2010).

37. D. Abebayehu, *et al.*, Polymer scaffold architecture is a key determinant in mast cell inflammatory and angiogenic responses: Scaffold architecture determines mast cell inflammatory response. *J. Biomed. Mater. Res.* **107**, 884–892 (2019).

38. M. Alksne, *et al.*, In vitro comparison of 3D printed polylactic acid/hydroxyapatite and polylactic acid/bioglass composite scaffolds: Insights into materials for bone regeneration. *Journal of the Mechanical Behavior of Biomedical Materials* **104**, 103641 (2020).

39. P. P. Spicer, *et al.*, Evaluation of bone regeneration using the rat critical size calvarial defect. *Nat Protoc* **7**, 1918–1929 (2012).

40. Department of Anatomy, Royal College of Surgeons in Ireland, 123 St. Stephens Green, Dublin 2, Ireland, J. Gleeson, N. Plunkett, F. O’Brien, Addition of hydroxyapatite improves stiffness, interconnectivity and osteogenic potential of a highly porous collagen-based scaffold for bone tissue regeneration. *eCM* **20**, 218–230 (2010).

41. G. Chen, C. Dong, L. Yang, Y. Lv, 3D Scaffolds with Different Stiffness but the Same Microstructure for Bone Tissue Engineering. *ACS Appl. Mater. Interfaces* **7**, 15790–15802 (2015).

42. N. Huebsch, *et al.*, Harnessing traction-mediated manipulation of the cell/matrix interface to control stem-cell fate. *Nature Mater* **9**, 518–526 (2010).

43. Y. Zhu, C. Goh, A. Shrestha, Biomaterial Properties Modulating Bone Regeneration. *Macromol. Biosci.* **21**, 2000365 (2021).

44. R. Vayron, *et al.*, Nanoindentation Measurements of Biomechanical Properties in Mature and Newly Formed Bone Tissue Surrounding an Implant. *Journal of Biomechanical Engineering* **134**, 021007 (2012).

45. R. Weinkamer, C. Eberl, P. Fratzl, Mechanoregulation of Bone Remodeling and Healing as Inspiration for Self-Repair in Materials. *Biomimetics* **4**, 46 (2019).

46. M. Haffner-Luntzer, A. Liedert, A. Ignatius, Mechanobiology of bone remodeling and fracture healing in the aged organism. *Innovative Surgical Sciences* **1**, 57–63 (2016).

47. K. Ishikawa, K. Hayashi, Carbonate apatite artificial bone. *Science and Technology of Advanced Materials* **22**, 683–694 (2021).

48. T.-I. Kim, J.-H. Jang, H.-W. Kim, J. Knowles, Y. Ku, Biomimetic Approach to Dental Implants. *CPD* **14**, 2201–2211 (2008).

49. N. López-Valverde, B. Macedo-de-Sousa, A. López-Valverde, J. M. Ramírez, Effectiveness of Antibacterial Surfaces in Osseointegration of Titanium Dental Implants: A Systematic Review. *Antibiotics* **10**, 360 (2021).

**Figure legends**

**Fig. 1. Synthesis of PLLA/gelatin floccular scaffolds for bone regeneration therapy.** (A) Schematic illustration of the fabrication process used to produce PLLA/gelatin floccular scaffolds, based on PLLA/gelatin floccular fibers, using emulsion electrospinning. (B) Photograph of as-formed floccular PLLA/gelatin fabrics on the target electrode and (C) cut PLLA/gelatin floccular fabrics. (D) Photograph of the PLLA/gelatin floccular scaffolds. (E-I) SEM images of the PLLA/ gelatin floccular scaffolds (E, 3DPL1) (F, 3DPL2), (G, 3DPL4), (H, 3DPL6) and 2D sheet scaffolds (I, 2DPL). Scale bar, 50 μm. (J) Fiber diameter distributions of the PLLA/ gelatin floccular fibers from LCM observations. (K) IR spectra of the PLLA/gelatin floccular fibers (i) and their chemically etched residue (ii) with IR spectra of the PLLA (iii) and gelatin (iv) included for comparison. (L) Tensile stress-strain curves of the floccular PLLA/gelatin nonwoven fabrics. (M) Degradation of the PLLA/ gelatin floccular scaffolds under a wet environment. (N) Compression stress-strain curves of floccular PLLA/gelatin nonwoven scaffolds (3DPL4).

**Fig. 2. HAOB-3DPL4 constructs fail to regenerate bone following** xenotransplantation into a micro-mini pig furcation defect model. (A) Overview of the timeline for defect preparation and HAOB-3DPL4 scaffold construct preparation for invitro osteogenic characterization and transplantation into a bone defect. (B) Osteogenic-related gene expression of HAOB-3DPL4 scaffold constructs after 14 and 21 days incubation under osteogenic medium. (C) Three-dimensional (3D) reconstruction of micro-CT images and axial view of the furcation defect following HAOB-3DPL4 constructs and 3DPL4 scaffold transplantation into the established micro-mini pig furcation defect model. (D) Representative sections of HAOB-3DPL4 constructs and 3DPL4 scaffold transplant samples were examined by hematoxylin and eosin staining. (E) Quantification of the regenerated alveolar bone in terms of bone volume, trabecular bone, and bone mineral density from reconstructed 3D micro-CT images. Representative sections of 3D4PL4 and HAOB-3D4PL4 scaffold constructs examined by hematoxylin-eosin (HE) and Masson’s trichrome (MT) staining. (ns; not significant).

**Fig. 3. Analysis of the osteoblast differentiation ability of MCOB.** (A) Representative images of alizarin red and ALP staining of MCOB at day 10 without (upper panel) and with (lower panel) ODM treatment. Relative positive alizarin and ALP activity detected only in MCOB conditioned with ODM. (B) The relative mRNA expression levels of Osterix, Osteocalcin, and Runx2 of MCOB treated with ODM for10 days were shown as fold change by Real Time- PCR analysis. (C) SEM images of MCOB seeded in 3DPL4 scaffold (left). Higher magnification (right) showing external cell-cell and cell-3DPL4 contact indicated by red arrows. (D) SEM images of 3DPL4 scaffold (left). Higher magnification (right) showing three dimensional arrangement of polylactic acid fibers. (E) The photographs of H&E staining of MCOB-3DPL4 and 3DPL4 (F) implant groups at 4 weeks (upper) and 8 weeks (lower) after the subcutaneous transplantation into mouse. Boxed areas are shown at higher magnification. Higher magnified view of MCOB-3DPL4 groups showed ectopic bone formation within 3DPL4 scaffold at both 4 and 8 weeks post implantation, highlighted by yellow arrow heads. Higher magnified view of 3DPL4 groups showed connective formation within 3DPL4 scaffold and yellow arrow heads indicate the polylactic (PL) fibers.

**Fig. 4. Bone regeneration capacity in the mice alveolar bone defect evaluated by microCT (μCT) and Histological analysis.** (A) 2Dimensional μCT images of the defect areas, filled with MCOB-3DPL4, 3DPL4, Cytrans and empty defect groups at 4 and 8 weeks post transplantation. (B) μCT quantification of new bone volumes in the mice alveolar defect areas. (C) three Dimensional reconstructed images of the defect area. (D) Representative Haematoxylin and Eosin (H&E)-stained images of the low (Upper row) and high (framed regions of upper row) magnifications of the defect areas at 8 weeks post transplantation. Yellow arrow heads indicate 3DPL4 fibers, red arrow heads indicate cytrans granules, M3: maxillary third molar, M2: maxillary second molar, M1 area: maxillary first molar area/defect area, Black arrow indicates mesial direction of the jaw, yellow dotted line represents the outer edge of the regenerated bone, blue dotted line represents cemento-enamel junction line (CEJ) and the area below it indicates bone defect area.

**Fig. 5. Biomechanical and functional properties of regenerated bone at 8 weeks post-transplantation**. (A) Hardness and (B) elastic modulus of regenerated bone at a maximum load. (C) Two dimensional and (D) three dimensional μCT images of a mouse alveolar defect area at 4 weeks post-implant placement. (D) HE staining of the mouse alveolar bone defect at 4 weeks after implant placement. Boxed regions are shown as higher magnified sections in middle and lower row. M2: maxillary second molar, M1 area: maxillary first molar area/regenerated bone area, black arrow indicates mesial direction of the jaw.
