# Supplementary material for: Correction of large jawbone defect in the mouse using immature osteoblast–like cells and a 3D polylactic acid scaffold
Source: PNAS Nexus. 2022 Aug 18;1(4):pgac151. doi: 10.1093/pnasnexus/pgac151 (PMC9802318; doi:10.1093/pnasnexus/pgac151)
Supplement: pgac151_Supplemental_Files [file pgac151_supplemental_files.zip › PNASNEXUS-PNASNEXUS-2022-00126-s02.docx]

**Correction of large jaw bone defect in the mouse using immature osteoblast-like cells and a three dimensional polylactic acid scaffold**

Shigeto Suzuki ^1*^, Venkata Suresh Venkataiah ^1*^, Yoshio Yahata ^1^, Akira Kitagawa ^1,2^, Masahiko Inagaki ^3^, Mary M. Njuguna^1^, Risako Nozawa^1^, Yusuke Kakiuchi ^1^, Masato Nakano ^1^, Keisuke Handa ^1,4^ Masahiro Yamada^5^, Hiroshi Egusa^5^ and Masahiro Saito^1,2^

1. Division of Operative Dentistry, Department of Ecological Dentistry, Graduate School of Dentistry, Tohoku University, Sendai, Miyagi 980-8575, Japan.
2. OsteRenatos Ltd. Sendai Capital Tower 2F, 4-10-3 Central, Aoba-ku, Sendai, Miyagi, 980-0021, Japan
3. National Institute of Advanced Industrial Science and Technology, 2266-98 Anagahora, Nagoya, Aichi 463-8560, Japan; [m-inagaki@aist.go.jp](mailto:m-inagaki@aist.go.jp)
4. Department of Oral Science, Division of Oral Biochemistry, Graduate School of Dentistry, Kanagawa Dental University, Yokosuka, Kanagawa 238-8580, Japan.

5. Division of Molecular and Regenerative Prosthodontics, Tohoku University, Graduate School of Dentistry, Sendai, Miyagi 980-8575, Japan

Corresponding author: Masahiro Saito, PhD. DDS

**Email:**  [masahiro.saito.c5@tohoku.ac.jp](mailto:masahiro.saito.c5@tohoku.ac.jp)

**This PDF file includes:**

Supplementary text

Figures S1 to S11

Table S1 & S2

Movie S1 and S2 legends

SI References

**Other supplementary materials for this manuscript include the following:**

Movies S1 and S2

**Supplementary information**

Extended material

**Materials and Methods**

**Fabrication of the 3DPL4 scaffold**

Poly L-lactic acid (i.v. = 0.8–1.2 dl/g; Sigma Aldrich) was dissolved in chloroform (Fujifilm Wako Pure Chemical Industries, Ltd.) at a weight ratio of 1:8. To increase the strength of the scaffolding material with respect to 9 g of this polymer solution, a solution containing 160 mg of gelatin (average M.W. = 100,000; Fujifilm Wako Pure Chemical Industries, Ltd.) in 2 mL of formamide (Sigma Aldrich) was added to prepare an emulsion. This emulsion was then spun into cotton-like fibers at an applied voltage of 1 kV/cm using an electrospinning apparatus (Kato Tech). The obtained PLA fabrics were cut to a size of 8 × 8 cm^2^, and a plurality of the obtained PLA fabrics were stacked and compression-molded to a thickness of 2 mm to create a fiber density. The porosity of the 3DPL4 scaffold was prepared to ca. 98%. The number of stacked PLA fabrics was used as the sample number (example: 3DPL4 when the number of stacked PLA fabrics was 4). After vacuum drying for 48 h to remove the residual solvent, the obtained molded product was cut with a laser cutting machine (Smart-Lab) to prepare a scaffolding material of 5 × 3 × 2 mm. The prepared scaffold material was treated with 10 mmol of EDC (1-ethyl-3- (3-dimethylaminopropyl) carbodiimide hydrochloride; Combi-Blocks) in 70% ethanol to crosslink the gelatin. Then the EDC treated scaffolds were rinsed three times with ultrapure-water and freeze-dried following pre-freezing at -30°C. The tensile mechanical properties of the prepared scaffolds were evaluated using an autograph-testing machine (EZ-SX, Shimadzu Corporation) operated at 1 mm/min under a 5-N load cell. For the tensile measurements, the test specimens were cut into dumbbell shape according to ISO 37 type 4 using a cutting blade (Dumbbell Co., Ltd.). A compression test was also performed with the tabletop tester on a 1 cm^3^ piece of scaffold material prepared so as to have a fiber density similar to that of 3DPL4, and the behavior of the material during compression deformation was evaluated.

**Preparation of HAOB, MCOB and 3DPL4, and transplantation protocol for mice maxilla bone defect model**

MCOBs were isolated and cultured from the calvaria of 1-week-old wild-type (WT) C57BL/6N mice following a previously described protocol (Aino et al 2014), and MCOB-3DPL4 constructs were prepared and transplanted into a maxilla large bone defect model in the mouse. Detailed information are available in SI Appendix regarding MCOB or HOAB isolation and the preparation of the 3DPLA complex, the mouse maxilla bone defect model, the transplantation of MCOB-3DPL4, osteogenic differentiation assays, minipig bone defect model and HAOB-3DPL4 transplantation, histological analysis, immunostaining, alizarin red and alkaline phosphatase staining, quantitative PCR analysis, quantitative bone mass analysis of new bone using µCT, nanoindentation analysis, mice implant therapy model analysis, and statistical analysis.

**HAOB isolation from the human alveolar bone**

With the approval of the Institutional Review Board of Tohoku University, human alveolar bone particles were collected after obtaining written informed consent from the study subjects (2018-3-024). HAOBs were isolated and cultured from healthy human alveolar bone as previously described (1). Briefly, surgically collected bone particles were placed in a sterile PBS solution containing antibiotics and transferred to the laboratory. The attached soft tissues from alveolar bone particles were removed and washed three times with PBS to obtain pure bone tissue under aseptic conditions. The bone tissue was minced into smaller-sized particles (1-2 mm3) and incubated in 4 ml collagenase solution (3mg/ml, Collagenase P; Roche Diagnostics, Germany) followed by sequentially digested in a water bath at 37°C to obtain a total of eight fractions. The collagenase digested solution of each fraction was diluted with DMEM containing 10% FBS and centrifuged. After subsequent centrifugation, the cell pellet from each fraction was cultured in a 6-well plate under an MF Start medium (Toyobo) supplemented with 1% penicillin/streptomycin, 0.25 μg/ml gentamicin and 10 ng/ml fungizone. These cells were passaged and maintained as HAOBs. Among the isolated HAOBs, cells from fraction five were utilized for further*in vitro* and *in vivo* experiments.

**HAOB proliferation on a 3DPL scaffold**

Prior to cell seeding, all scaffolds to be used in the experiments were sterilized by immersion in 70% ethanol for 1 minute, followed by four washes in PBS for 1 minute each to completely remove the ethanol. The scaffolds were then soaked in a culture medium for 1 hour before cell seeding. 96 well non-adherent tissue culture plates were used (Falcon) to facilitate preferential cell attachment to the scaffolds. The cultured HAOBs to be seeded onto the scaffolds were first trypsinized in 0.05% trypsin/0.02% EDTA (Gibco, 25200-056), and the detached cells were pelleted by centrifugation at 1500 rpm for 5 min and then resuspended in MF culture medium, supplemented with 1% FCS. 10 µl cell suspensions containing 5x103 HAOBs in MF medium were seeded onto 2DPL, 3DPL and collagen scaffolds which were then immediately placed in a humidified atmosphere containing 95% air and 5% CO2 for 2 hours to facilitate cell attachment; 100ml of culture medium was then added to each well. Cell proliferation was assayed using a Cell Counting Kit 8 (CCK 8; Dojindo Molecular Technologies) following the manufacturer's protocol on days 1, 4, 7, 14 and 21. Cell proliferation was next evaluated on the 3DPL scaffolds of varying stiffness (3DPL1, -2, -4 and -6) using the same protocol described above on days 1, 3 and 7.

**Water contact angle of 3DPL Scaffold material**

In general, the contact angle of a material surface is greatly affected by surface roughness. In the case of fibrous porous materials, especially those containing an internal gas phase, the apparent contact angle is known to be larger than the true contact angle. To avoid this problem, a plate sample with a flat surface was prepared by placing the 3DPL scaffold between glass slides, compacting at 1800 C, and carefully removing the glass slides after cooling. The raw polymer powder was also processed in the same way to form a plate for comparison. Three microliters of milli-q water were dropped on the surface of the scaffolds. An optical contact angle meter (DM300, Kyowa interface science Co., Ltd., Japan) was used to analyze the contact angle of a water droplet on a sample surface. The contact angle was estimated by a curve fitting method for digital images of water droplets using analysis software (FAMAS, Kyowa interface science Co., Ltd., Japan). The surface morphology and surface roughness of the samples were measured using a laser confocal microscope (LEXT OLS 3000, Olympus, Japan)

**Alizarin red and alkaline phosphatase staining**

The extracellular matrix calcification of formalin fixed HAOB-seeded 3DPL scaffolds and MCOB cells were evaluated by staining with 2% alizarin red solution (Wako) for 10 sec and 5 min, respectively. ALP activity in the HAOB-seeded 3DPL scaffolds and MCOB cultures was detected using an ALP staining solution consisting of 0.1 mg/ml naphthol AS-MX phosphate (Sigma Aldrich, St. Louis, MO), 0.6 mg/ml Fast Blue B salt (Sigma Aldrich), 2 mmol/L MgCl2 (Wako), 0.5% N, N-dimethylformamide (Wako) and Tris-HCl (pH 8.5) at room temperature for 10 sec and 20 min, respectively.

**HAOB-3DPL4 construct preparation for osteogenic characterization and transplantation**

 3DPL4 scaffolds were sterilized as described above. 20 µl cell suspensions containing 5x10^5^ HAOBs were seeded onto each scaffold via a droplet method in a 24-well non-adhesive tissue culture plate (Falcon) to promote the attachment of the cells to the scaffold. To determine the most appropriate osteogenic stimulant for the HAOBs seeded 3DPL4 scaffold, these constructs were cultured in basal medium (MF medium) for one week and then treated with osteogenic differentiation medium (ODM) comprising MF medium supplemented with 10 mM β-glycerophosphate (Sigma Aldrich), 50 μg/ml ascorbic acid (Sigma Aldrich), 10 nM dexamethasone (Wako), and the following osteogenic stimulants either alone or in combination for two and three weeks: SAG at 1 nM (Calbiochem), TH at 1 nM (Takeda Pharmaceutical Company Limited), SAG + TH, BMP2 at 100ng/ml (recombinant human bone morphogenetic protein (rhBMP)-2; R&D systems). HAOB seeded 3DPL4 scaffold constructs without ODM treatments served as a control group. Real-time PCR was performed to analyze the expression of osteogenic related genes. For transplantation into micro-mini pig furcation defects, the HAOB-seeded 3DPL4 scaffolds were cultured in basal medium for one week and then in osteogenic medium (50 mg/mL ascorbic acid phosphate (Sigma Aldrich), 10 mM β-glycerophosphate (Sigma Aldrich), 10 nM dexamethasone (Wako), 1 nM SAG (Calbiochem), 1nM TH (Takeda Pharmaceutical Company Limited)) for two weeks.

**Minipig bone defect model and HAOB-3DPL4 transplantation**

 Minipig bone defects were prepared as described previously (2). Briefly, all surgical procedures were performed under general anesthesia (sevoflurane) and local anesthesia with 2% lidocaine (Aura Injection, Showa Yakuhin Kako) administered for intraoperative pain management. Opioid analgesics (repetan suppository 0.4 mg, Otsuka Pharmaceutical) were used for postoperative pain management. Furcation defects were generated in the mandibular second premolar on the right and left sides of the jawbones. These defects were prepared using sterile carbide burs with a low-speed handpiece under continuous sterile saline irrigation. The size of the defects (7 mm height, 5 mm deep and 5 mm wide) standardized using a periodontal probe, and each defect was filled with silicone impression paste (Tokuyama Dental Corp, Tokyo, Japan) to retain the original size of the defect. Reference notches were placed on each root to indicate the basal level of the defect. One month after defect preparation, the silicone impression material from the defect area was removed, disinfected with isodine, and washed thoroughly with saline. The right side of the defects was then filled with the HAOB-3DPL4 constructs and left side with a 3DPL4 scaffold without cells. The minipigs sacrificed and mandibles were collected after two months of transplantation and fixed in 10% formalin for further analysis.

**MCOB isolation**

Experiments in mice conducted with the approval of the National University Corporation Tohoku University Environment and Safety Committee Animal Experiment Special Committee in compliance with the "Regulations on Animal Experiments" at Tohoku University "(2019-Shidou-062-02) ". Wild-type (WT) C57BL / 6N mice were purchased from SLC Japan and in the Animal Care and Use Facility of Tohoku University. MCOBs were isolated and cultured from the calvarias of 1-week-old wild-type (WT) C57BL / 6N mice following the previously-used protocol for HAOB isolation. Briefly, collected calvarias were sequentially digested using a bacterial collagenase (Collagenase P; Roche Diagnostics, Germany) dissolved in PBS. MCOBs were isolated and cultured in the same manner as HAOBs, and the cells from fraction three were used for further experiments.

**Anesthetic procedures in the mouse experiments**

Five-week-old healthy wild-type (WT) C57BL/6N mice were used in the MCOB-3DPL4 autotransplantation experiments. All surgical procedures were performed under a surgical microscope (Carl Zeiss) following intraperitoneal injection of anesthetic solution containing 1 mg/ml medetomidine hydrochloride, 5 mg/ml midazolam, 5 mg/ml butorphanol tartrate.

**MCOB-3DPL4 construct preparation**

MCOB-3DPL4 constructs were prepared in a similar manner to the HAOB-3DPL4 constructs with minor modifications. Cells were seeded at a density of 3.3x10^4^ cells/scaffold and incubated for two hours to allow for attachment prior to transplantation.

**Live/dead Assay of MCOB seeded 3DPL4 scaffold constructs**

Cell-scaffold complexes were prepared as described in MCOB-3DPL4 construct preparation. After 1 and 3 days of in-vitro culture, Live/dead staining of MCOB seeded onto 3DPL4 scaffolds was evaluated using a Live/dead viability/cytotoxicity test kit for mammalian cells (Invitrogen, L3224) following the manufacturer's protocol. Briefly, on the day of assay, MCOB-3DPL4 scaffold constructs were treated with the assay solution consisting of 4 μL of 2 mM ethidium homodimer-1 (EthD-1) and 1ul of 4mM calcein-AM in 2ml PBS for 30 minutes at room temperature. Cell viability within the 3DPL4 scaffolds was observed and imaged by a Zeiss LSM800 Confocal Microscope.

**Preparation of a mouse alveolar bone defect model and transplantation**

To generate the large jawbone defect model, the maxillary right first molar was extracted by luxation using a needle tip (20Gx1.1/2, Terumo), and a small sized needle holder was used to remove the tooth by a gentle back and forth motion without damaging the alveolar bone. After four weeks of the socket and soft tissue healing, bone defects were prepared using a Meisinger Steel Bar ST1RA005 (Heleus Kurzer, Japan) in the maxillary first molar area (M1area). The size of the bone defect was 1 mm mesiodistally, 0.5 mm in a buccolingual width and 0.3 mm deep. The defects were then filled with the following groups: 1) MCOB-3DPL4 constructs, 2) 3DPL4 scaffold without cells, 3) Cytrans (carbonate apatite Granules S size, GC). Empty (unfilled) defects were used as a comparison control. All the defects were then sutured using an 8-0 suture (bio-fit; WASHIESU). Following transplantation, a non-steroidal anti-inflammatory drug (Inflacam, Virbac) was administered to relieve pain and reduce inflammation. The mice maxillary jaw was collected four and eight weeks after transplantation for further analysis.

**Histological analysis of the mouse jawbones**

 Collected jawbones from the experimental mice were fixed with 10% formalin for three days, treated with decalcification solution (Kalkitox: Fujifilm Wako Pure Chemical Industries, Ltd.) for five days, and embedded in paraffin. Continuous thin specimens of 5 μm in thickness were prepared in the buccolingual direction and then deparaffinized and stained with hematoxylin-eosin (HE). New bone regeneration was evaluated under an optical microscope (DM6000B; Leica, Germany).

**Immunostaining**

 For immunostaining analysis, formalin-fixed, paraffin-embedded specimens were placed on glue-coated glass slides. Briefly, the samples were deparaffinized in xylene and hydrated in a graded alcohol series and distilled water. Endogenous peroxidase activity was blocked with 3% hydrogen peroxidase for 10 min. Antigen retrieval was performed in citrate buffer (pH 9.0) by autoclaving at 121°C for 5 min (Tomy SX-500 High-Pressure Steam Sterilizer; Tomy Seiko Co., Ltd., Tokyo, Japan). The samples were then treated for 30 min at room temperature (RT) in a blocking solution with 1% rabbit serum (Nichirei Bioscience, Tokyo, Japan) followed by incubation with primary antibodies, osteocalcin (Takara Bio Inc) and anti-CD31 (R & D Systems) for 16 h at 4°C. Sections were then incubated with secondary antibody using an Envision + System-HRP labelled polymer anti-mouse antibody for 30 min at RT. DAB (3, 3 -diaminobenzidine) staining was used to visualize the binding of the first antibody, and these signals were detected using a Leica DM6000B microscope (Leica, Wetzlar, Germany).

**Quantitative real-time RT-PCR**

 Total RNA was isolated from cultured HAOB-seeded 3DPL4 scaffolds and MCOBs using the Nucleospin RNA kit (Macherey Nagel, Germany) following the manufacturer's instructions. cDNAs was synthesized from 1 μg of total RNA in a 20 µl reaction containing 10X reaction buffer, 1 mmol/L of dinitrophenol phosphate (dNTP) mixture, 1 U/ L RNase inhibitor, 0.25 U/ L reverse transcriptase (Invitrogen, Carlsbad, CA), and 0.125 mol/L random hexamers (Takara, Tokyo, Japan). The cDNAs were then amplified using human-specific primers (Osterix, Runx2, Bsp, Type1 collagen) and mouse-specific primers (Osterix, Runx2, Osteocalcin) using a real-time PCR apparatus (Bio-Rad CFX Connect; Applied Biosystems). The amplification conditions were 40 cycles at 95°C for 3 mins, 55°C for 30 sec, and 65°C for 5 sec. The expression of the tested osteogenic genes was calculated using the 2−ΔCT method and these values were compared with those for the GAPDH housekeeping gene. The sequences of the human and mouse primers used are listed in Supplementary Table 2.

**Quantitative micro-CT analysis of new bone in the defect area**

 The jawbones from the minipig bone defect and mice alveolar bone defect models were examined by micro-computed tomography (μCT) scanning following the post-transplantation period. Three-dimensional (3D) image analysis software (TRI/3D-BON; Ratoc System Engineering; and Amira; Thermo Scientific-JP) was used to measure the quantitative analysis of regenerated bone in the defect area of minipigs and mice bone defect models.

**Analysis of mechanical properties in new bone using nanoindentation test**

 Vickers hardness and Young's modulus were measured using the IBIS nanoindentation system (IBIS, Australia) with diamond Berkovich chips to assess the mechanical properties of the regenerated bone. The maxillary mouse jaws were embedded in cold epoxy resin (Zeromer K Plus, IMT) and were sliced using a diamond saw (Micro cutter MC-201, Marteau) in the mesiodistal direction to expose the defect area. The defect area was then ground using water-resistant abrasive paper (# 1500, # 1000) to expose the interior of the defect area and was further smoothened by polishing with a diamond bead suspension (9 μm, 3 μm, 1 μm). After processing, the sample surface was cleaned with an ultrasonic cleaner and used for nanoindentation measurements. In the nanoindentation test, the maximum load (Pmax) was set to 10 mN and the initial load to 0.015 mN. This load was applied at a constant speed to minimize the effects of viscoelastic deformation of the sample. A measurement method was used in which the load was held at the Pmax for 5 sec and then unloaded under the same conditions as the applied load. The indentation interval was 10 μm, and a total of 16 points were measured at room temperature. The samples used for the nanoindentation test was air-dried and then platinum-deposited, and indentations were confirmed with a scanning electron microscope (JSM-6390LA, JEOL Ltd.). The imaging conditions of the scanning electron microscope were 10 kv, 2000 times, and 5000 times.

**Functional analysis of regenerated bone using titanium implants**

 To evaluate the functional properties of the regenerated bone, titanium mini-implants/screws were placed into the defect area. Mini Implants (manufactured by Matsumoto Sangyo, Inc) with a length of 1.5 mm and a diameter of 0.8 mm, were used (3). Implant placement was carried out by creating an implantation drill using # 60 and # 70 ZIPPERER K files (VDW, Germany) at the center of the defect/regenerated bone area. The implants were fixed in the position using a mini implant holder. Histological and 3D imaging analysis performed after four weeks of implantation to evaluate the levels of osseointegration, and the implant survival rates.

**Statistical analysis**

Statistical comparisons of the bone masses in each group in the MCOB-3DPLA transplantation experiments using the mouse alveolar bone defect model (calculated as mean values with standard deviation), were conducted using one-way analysis of variance (ANOVA). Subsequent comparisons were made using Dunnett's multiple comparison test.

The mean values of bone Vickers hardness and elasticity in the nanoindentation test from each indent point from individual mice among the experimental group were compared by three-way ANOVA and Tukey's HSD test. The data processing program "JMP Pro 15" (SAS Institute Inc, Cary, NC, USA) was used with the significance level set at alpha = 0.05.

**Results:**

**Water contact angle measurement of the 3DPL scaffolds**

The hydrophilicity of the scaffolds used for bone tissue engineering plays a critical role in interacting with cells. The resultant water contact angle measurements of 3DPL scaffold materials (3DPL1, 2, 4 and 6) were 69.7^0^ (Fig. S3*A)*, and raw PLLA was 65.2^0^ (Fig. S3*B*). The water contact angle of 3DPL and raw PLLA material were less than 90^0^, indicating that these scaffolds are hydrophilic and allow direct cell seeding onto the scaffolds enhancing cell adhesion ability which is highly desirable for tissue engineering applications. The contact angle depends on the surface morphology. Fig. S3*C* and Fig. S3*D* reveal the appearance of a smooth surface on 3DPL and raw PLLA samples, ignoring the effect of porosity, thereby producing more reliable contact angle measurements of the scaffold materials. The surface morphology of the samples was confirmed by LCM observation (Fig. S3*E* & S3*F*) and showed that these samples have a roughness of submicron scale (Supplementary table 1), indicating the smooth surface of the materials used for contact angle measurements. A summary of the calculated contact angle and surface roughness measurements is shown in Supplementary table1.

**Cell proliferation ability of HAOB on different 3DPL scaffolds**

Scaffolds designed for tissue engineering need to be biocompatible with the regenerating cells and provide a suitable environment for attachment, proliferation and differentiation. The viability and proliferative ability of HAOBs were assessed on our 3DPL scaffolds using a CCK-8 assay at different time points. 2DPL and collagen sponge scaffolds were used as a control group. The results showed that cells in the 2DPL and 3DPL scaffolds had a comparable proliferative ability from day 1 to 21. Notably however, the cell growth in the collagen scaffold showed an initially high proliferation until day 4 and then a drastic decline from day 7 to 21 due to scaffold degradation (Fig. S4*A*). A far greater biocompatibility of the 3DPL scaffold for cell proliferation was thereby observed, and we evaluated HAOB proliferation ability on our series of these scaffolds with different stiffness levels, using the 2DPL scaffold as a control. The CCK-8 assay demonstrated HAOB cell growth in the 2DPL scaffold rapidly increasing from day 1 to day 7. In contrast, the HAOB cell growth on all the 3DPL scaffolds with varying stiffness showed a similar proliferation pattern, i.e., the cells actively proliferated until day 4 and then showed slow constant growth until day 7, except for 3DPL6 on which decreased growth was evident during this period (Fig. S4*B*).

**Invitro osteogenic differentiation ability of HAOB on different 3DPL scaffolds**

Osteoblastic differentiation ability of HAOBs seeded onto our series of 3DPL scaffolds (3DPL1, 3DPL2, 3DPL4, and 3DPL6) and 2D scaffold controls using alizarin red staining for mineralization ability and alkaline phosphatase (ALP) staining as an early osteoblast differentiation marker was investigated. The results demonstrated that 3DPL scaffolds stained more strongly for alizarin (Fig. S5*A*) and ALP (Fig. S5*B*) than 2DPL scaffolds at a range of different timepoints. Acellular 2D and 3DPL scaffolds showed weak alizarin staining (Fig. S5*A* lower row). Among the 3DPL scaffolds into which the cells had been seeded, 3DPL4 showed the greatest staining intensity for both alizarin and ALP, indicating its appropriateness as a scaffold for mineralization and osteoblastic differentiation (Fig. S5). It was thus determined that the 3DPL4 scaffold best mimicked the natural extracellular matrix and provided the most suitable microenvironment for the attachment, proliferation, and differentiation of osteoblasts. We used 3DPL4 as the cell carrier material in all our subsequent experiments.

**Cell viability of cultured MCOB on 3DPL4 scaffolds**

Viable cells within the scaffold are required for bone regeneration, whereby cells secrete extracellular matrix or growth factors to induce new bone formation.

Therefore, a Live dead assay was performed to assess the viability of MCOBs on the seeded 3DPL4 scaffolds. Live Cells were stained in green by calcein-AM and dead cells with red fluorescence by EthD-1 (Fig. S7). The number of live cells was increased from day 1 to day 3 with few random dead cells, indicating that the MCOB adhered and proliferated well on the 3DPL4 scaffold. The number of cells appeared uniformly distributed throughout the scaffold by day 3. The three-dimensional (3D) images show that MCOB were able to infiltrate within the 3PL4 scaffold and the cells are alive. Overall, the assay results indicated good biocompatibility of the 3DPL4 scaffold.

**Immunohistochemical analysis of blood vessels and osteocalcin in the regenerated bone**

Immunohistochemical analysis of the defect area for selected bone markers to evaluate active remodeling processes around the regenerated new bone was investigated. We analyzed by immunostaining the secretion of the bone matrix protein osteocalcin (OCN), a mature osteoblast marker, and CD31, a blood vessel marker for evaluating angiogenesis, in the regenerated bone at 8 weeks post-transplantation. Strong immunostaining of OCN was evident in both the MCOB-3DPL4 and cytrans groups. OCN staining was weak however in the empty group and was not detected in the 3DPLA group (Fig. S10*A*, lower panel). CD31-positive flat-shaped capillary vessel cells were detected in the regenerated bone in the MCOB-3DPL4, cytrans and empty defect groups (Fig. S10*B*, arrowheads), but none were observed in the 3DPL4 alone group.

**Nanoindentation analysis of regenerated bone**

Representative load-displacement depth curves of newly formed bone at 8 weeks post-transplantation revealed a typical nanoindentation load-depth along the longitudinal axis of the regenerated bone and indicated a lower penetration depth in the cytrans group followed by the 3DPL4, MCOB-3DPL4 and empty groups under the same maximum load (Fig. S12). The nanoindented measurements were taken from the center of the defect area in the MCOB-3DPL4, cytrans and empty defect groups (Fig. S13*A*). In the 3DPL4 group, the base of the defect, which represents native bone tissue was indented due to the absence of regenerated bone in the defect area (Fig. S13*A*, upper right panel).

A total of 16 indents (4 x 4 matrices) were acquired in each region of interest and the distance between them was10 μm in both the x and y directions. After completing the nanoindentation test, the specimens were coated with platinum using an ion sputter and the microstructures of the indentations were observed and photographed by SEM to confirm the nano indented points. (Fig. S13*B,* upper panel). Higher magnification observations of the nanoindented points in the region of interest are shown in Figure S13*B,* lower panel.

**Measurement of Bone implant contact ratio**

Quantification of the bone implant contact ratio (BIC) showed stronger osseointegration in the MCOB-3DPL4 and cytrans group. These BIC differences in the MCOB-3DPL4 and cytrans groups compared to the 3DPL4 and empty defect groups were significant, but not between the MCOB-3DPL4 and cytrans groups (Fig. S15).

Side view


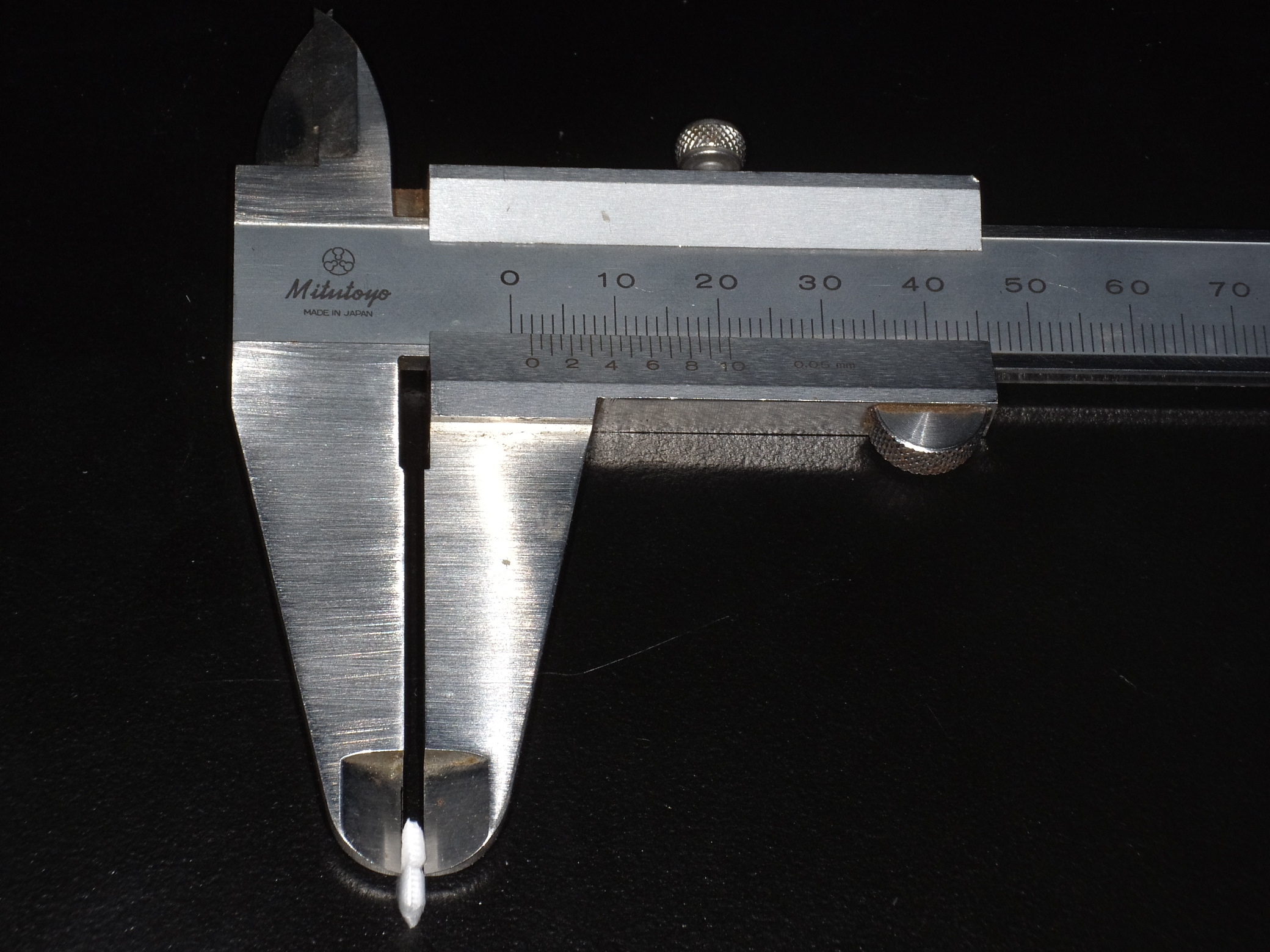


**Fig S1: Measurement of rectangular 3DPL4 scaffold.** Side view (thickness) of the scaffold measured by vernier caliper.

**
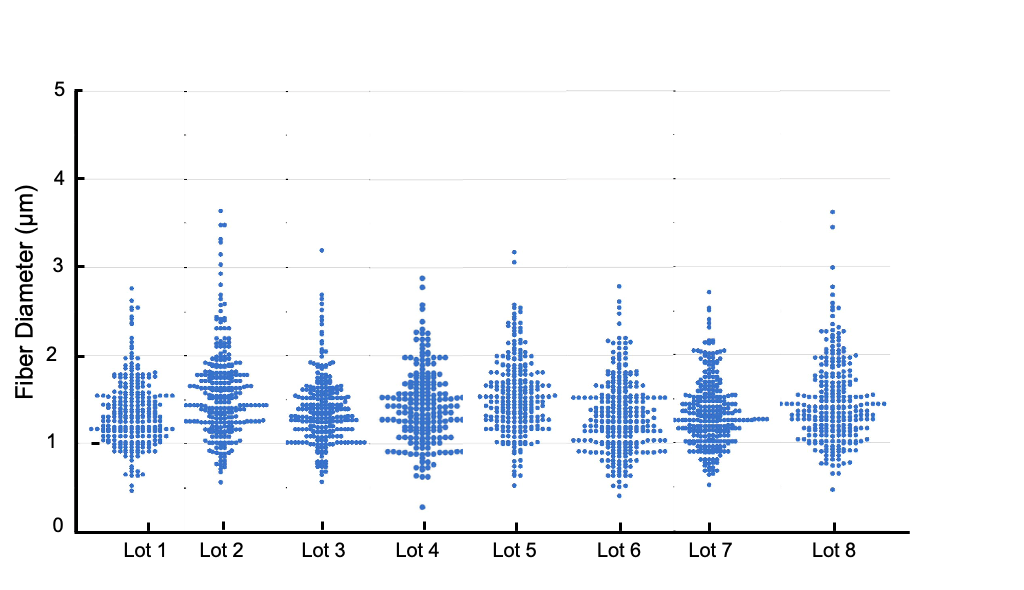
Fig S2.** Fiber Diameter distribution of eight lots of PLLA/gelatin floccular fibers by Laser confocal microscope (LCM).


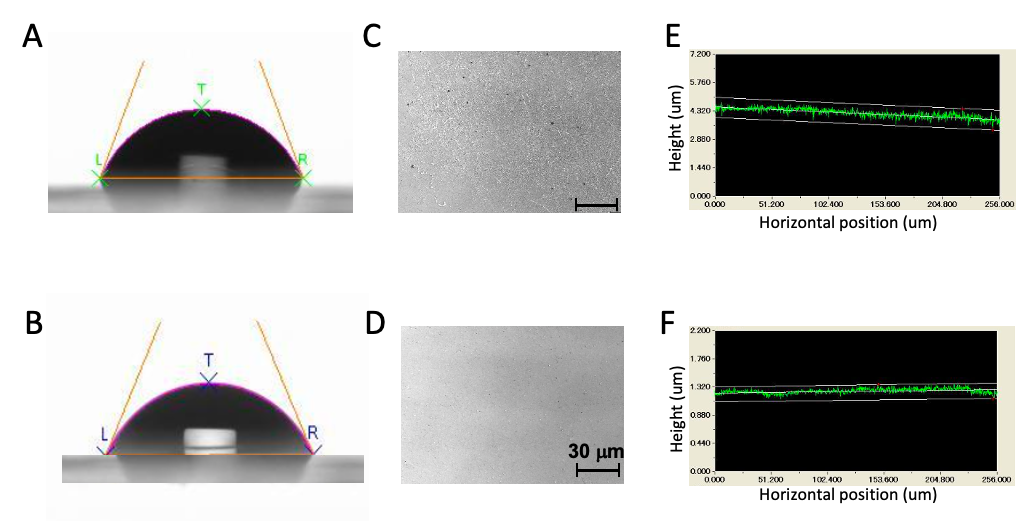


**Fig. S3. Water Contact angle measurements of the PLA scaffolds.** Contact angle of 3DPL (A) and raw PLLA (B) pressed at 180^0^ C. Surface morphology of 3DPL (C) and raw PLLA (D) specimens used for contact angle measurement. Cross sectional curves of 3DPL (E) and raw PLLA (F) specimens used for measuring average surface roughness.

**
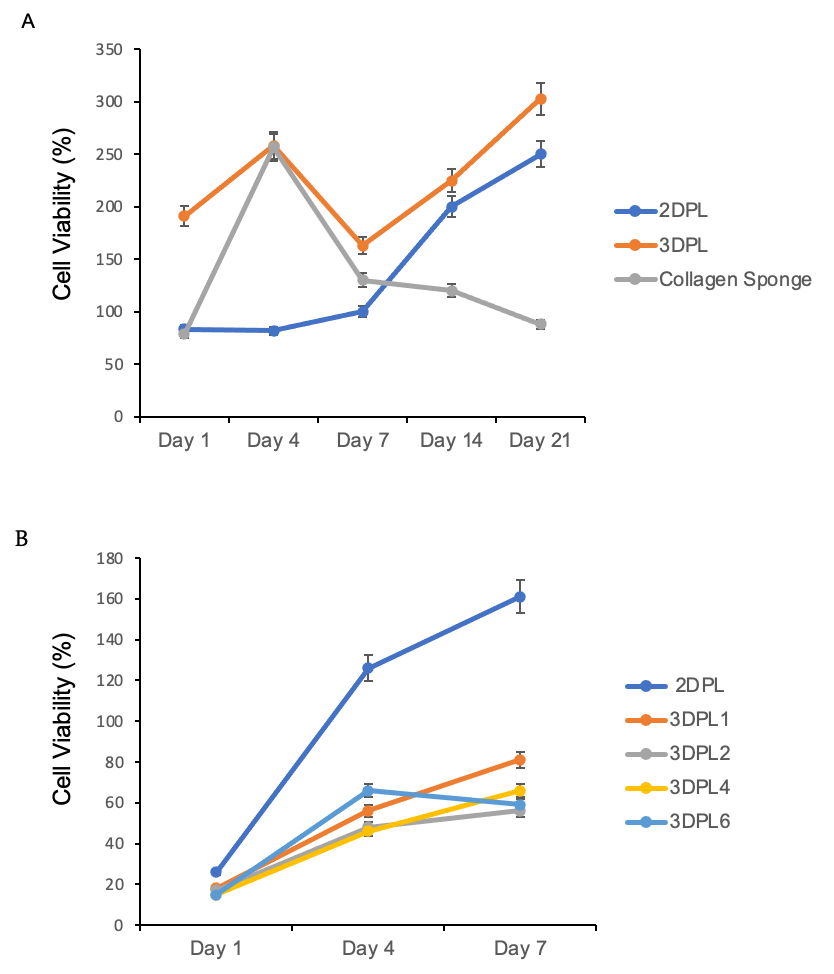
**

**Fig S4. HAOB cell proliferation within 3DPL scaffolds to assess cell viability**. (A) HAOB cell proliferation was measured on 3DPL, 2DPL, and collagen sponge scaffolds at the indicated timepoints. (B) HAOB cell proliferation on 2D, 3DPL1, 3DPL2, 3DPL4, and 3DPL6 scaffolds after 1, 3 and 7 days of culture. The relative cell viability was calculated as the percentage of total live/total cells.


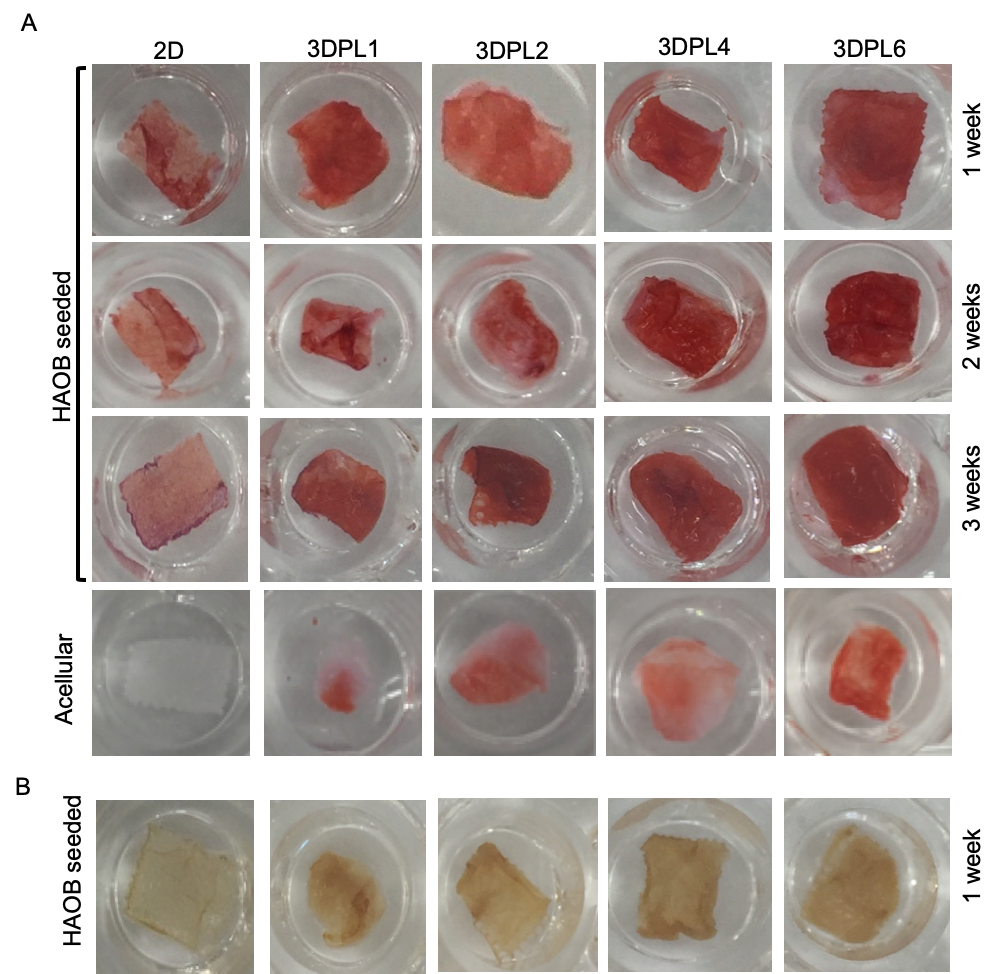


**Fig S5. In vitro osteogenic differentiation ability of HAOB-seeded 3DPL scaffolds.** (A) Alizarin red staining images of HAOB-seeded 2D, 3DPL1, 3DPL2, 3DPL4, 3DPL6 scaffolds maintained in osteogenic medium for 1, 2 and 3 weeks in comparison with acellular scaffolds. (B) Alkaline phosphatase staining of HAOB-seeded 2D, 3DPL1, 3DPL2, 3DPL4, 3DPL6 scaffolds maintained in osteogenic medium for 1 week.

**Fig. S6.** Schematic experimental overview and timeline for *in vivo* orthotopic bone regenerative ability of the MCOB-3DPL4 constructs.


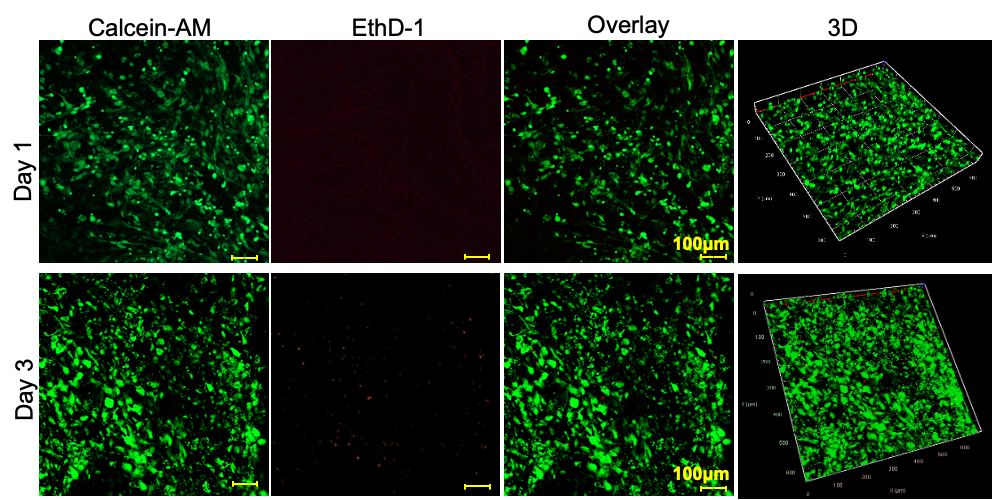


**Fig S7.** **Cell viability assay of MCOB-3DPL4 scaffold constructs**. Representative confocal images of MCOB seeded on 3DPL4 scaffold at 1 and 3 days of culture. Alive and dead cells were stained with green and red fluorescence respectively. [3D: three dimensional].


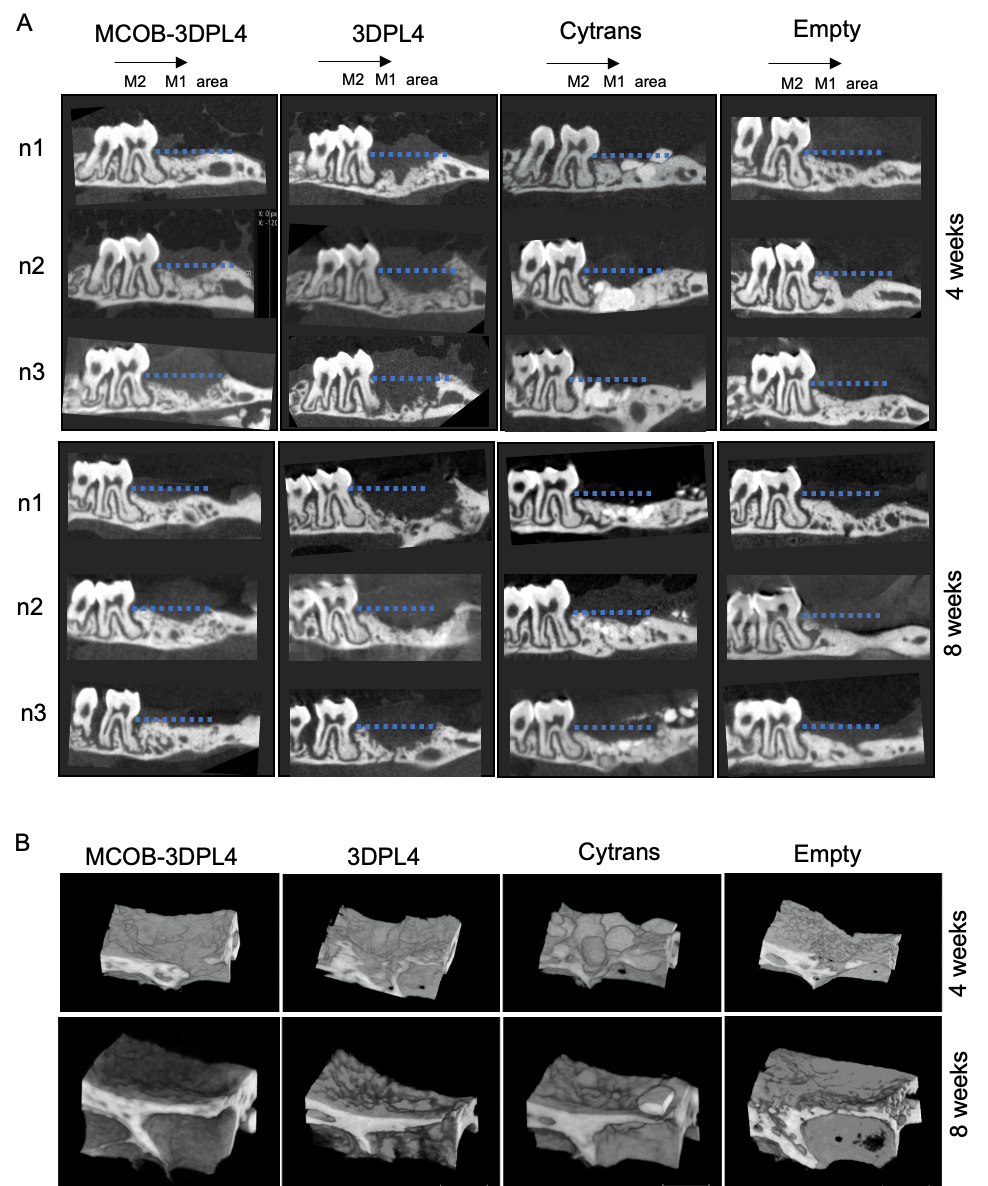


**Fig. S8. Micro-computed tomography images of the mouse alveolar bone defect defect area following transplantation.** Representative two-dimensional images (A) and three-dimensional constructed images (B) of μCT data of regenerated bone in a mice alveolar bone defect model of all the samples at 4 and 8 weeks post transplantation. M2: maxillary second molar, M1 area: maxillary first molar area/defect area, Black arrow indicates mesial direction of the jaw, blue dotted line represents cemento-enamel junction (CEJ) and the area below it indicates bone defect area.


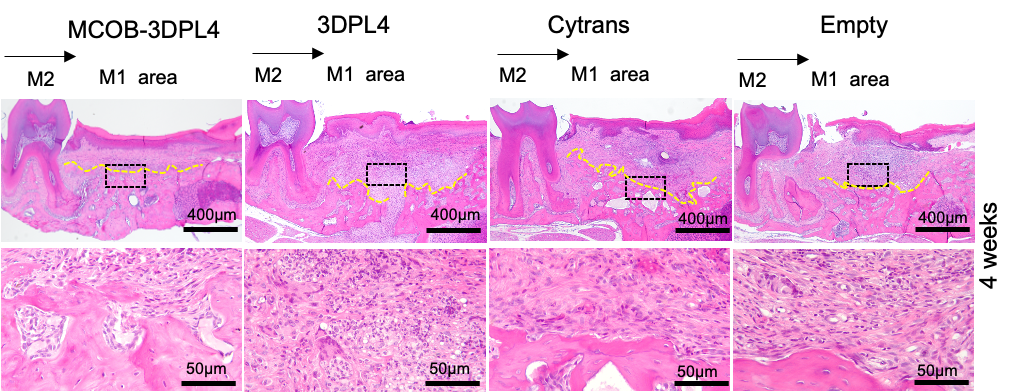


**Fig. S9**. **Histological evaluations of the bone regenerative capacity of the MCOB-seeded-3DPL4 scaffold in the mouse alveolar bone defect model.** Upper panel show representative histological images of the alveolar bone defect areas in the mice at 4 weeks post-transplantation, visualized by HE staining. The lower panel show a high-magnification view of the boxed areas. M2: maxillary second molar, M1 area: maxillary first molar area/defect area, black arrow indicates mesial direction of the jaw, dotted yellow line represents the outer edge of the regenerated bone.


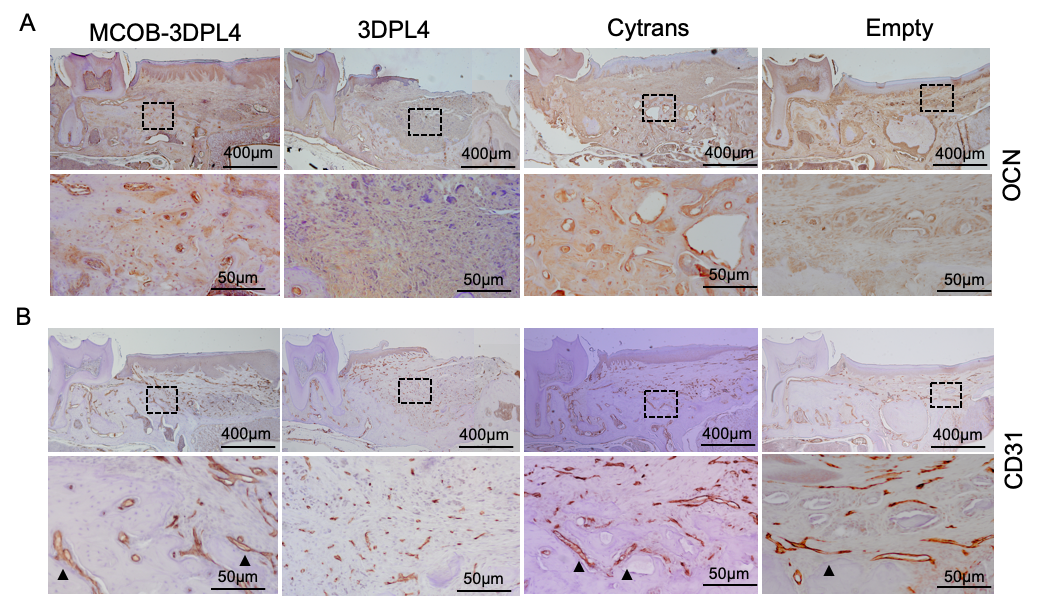


**Fig. S10. Immunohistological analysis of alveolar bone defect areas at 8 weeks post-transplantation in the mouse defect model.** (A) The defect areas in each experimental group were immunohistologically stained with an anti-osteocalcin antibody (OCN) and (B) anti-CD31 antibody. The lower panel of A and B show a high-magnification view of boxed areas from the upper panel.


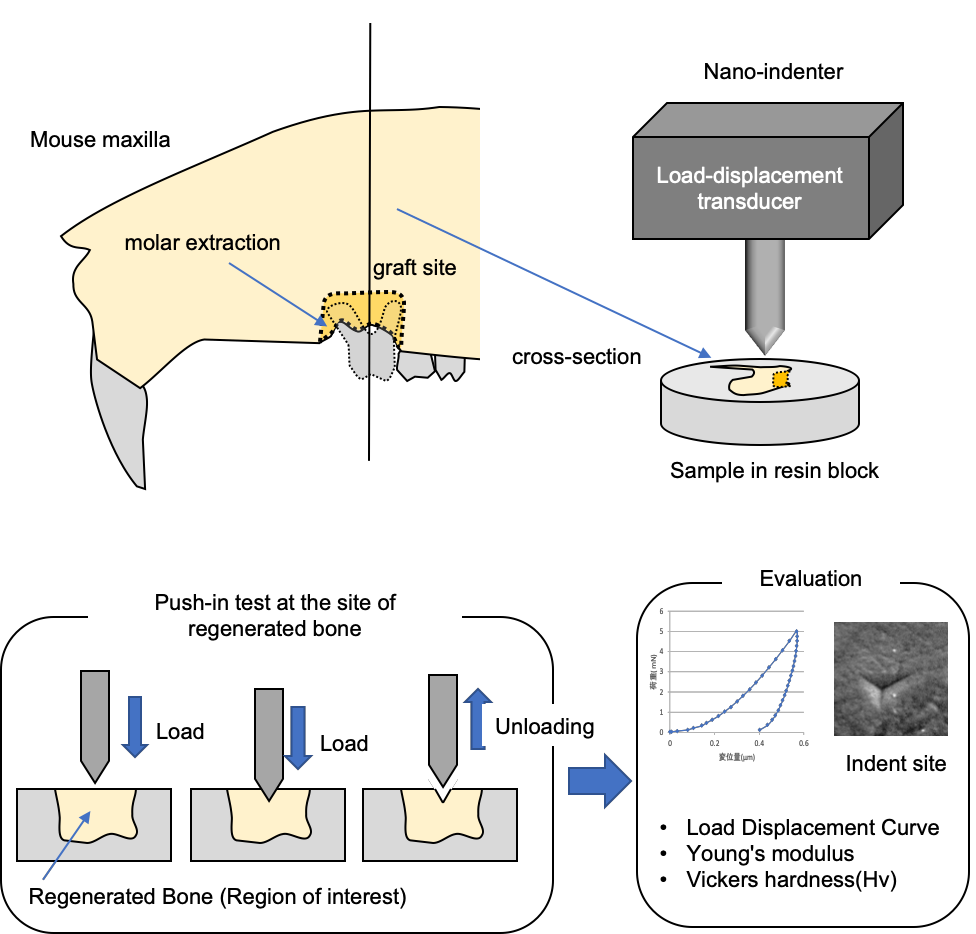


**Fig. S11. Schematic outline of nanoindentation test to determine the mechanical properties of the regenerated bone.**


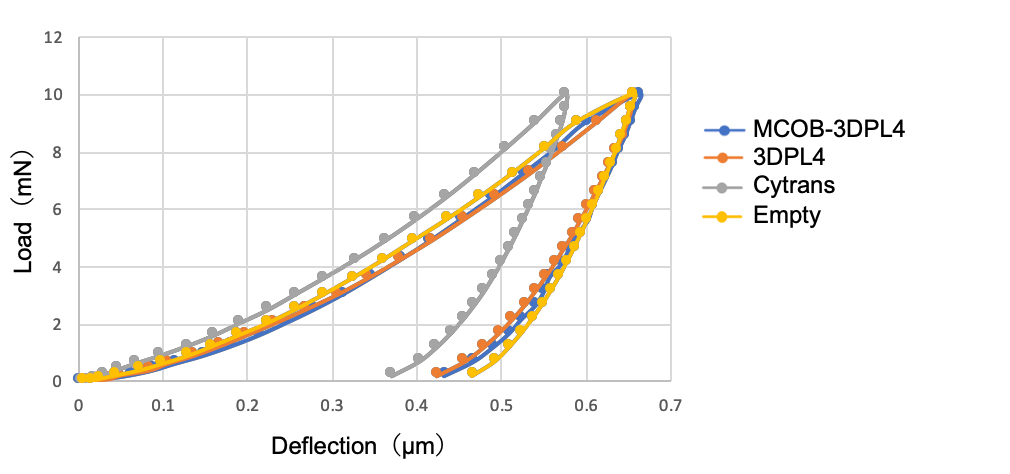


**Fig. S12. Typical load-displacement data of regenerated bone along the centre of the defect area.**


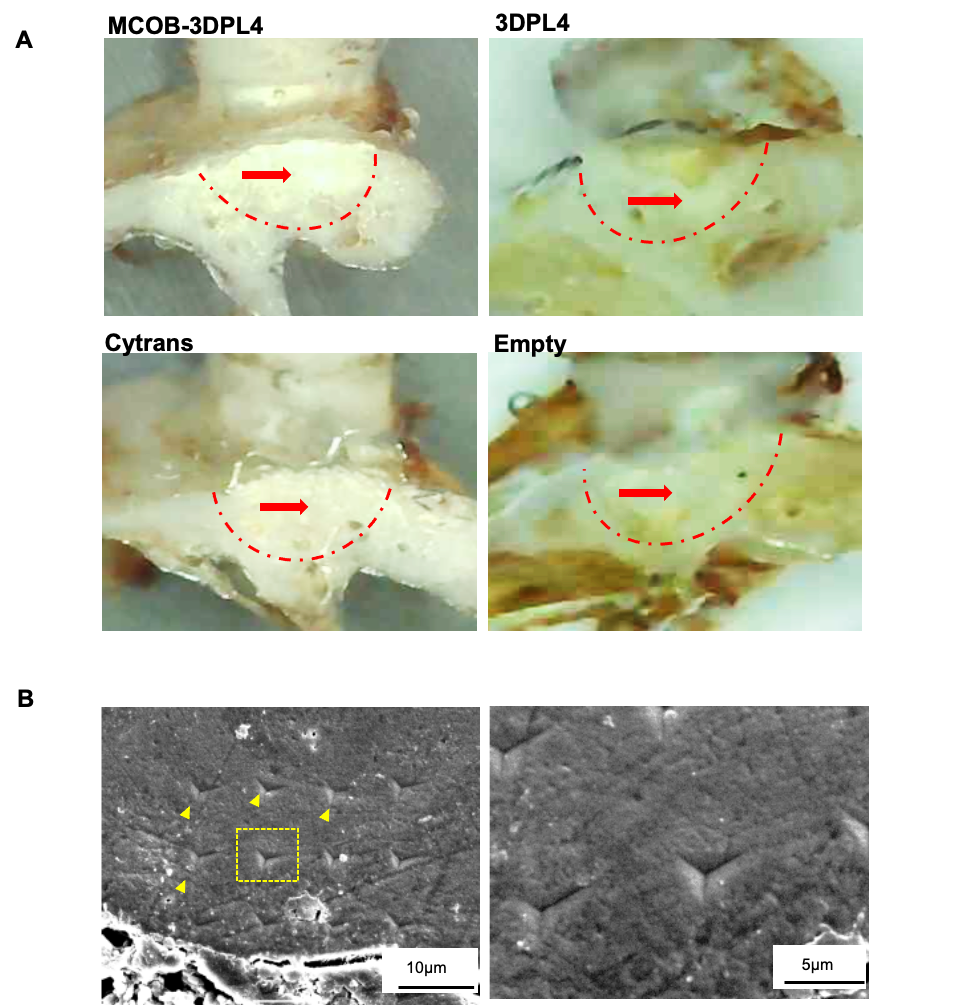


**Fig. S13. Nanoindentation evaluation of regenerated bone from the mice alveolar bone defect area.** A) Representative images showing the area undergone nanoindentation tests. The red arrow indicates the indented part within the regenerated bone except for 3DPL4, an intact bone in base of bone defect was indented which represents native bone tissue, and the dotted line represents the base of bone defect. B) An representative SEM image showing the indentation of nonindented region in regenerated bone (left panel, arrow heads). Boxed region in the left panel image is shown a higher magnified view in the right panel.


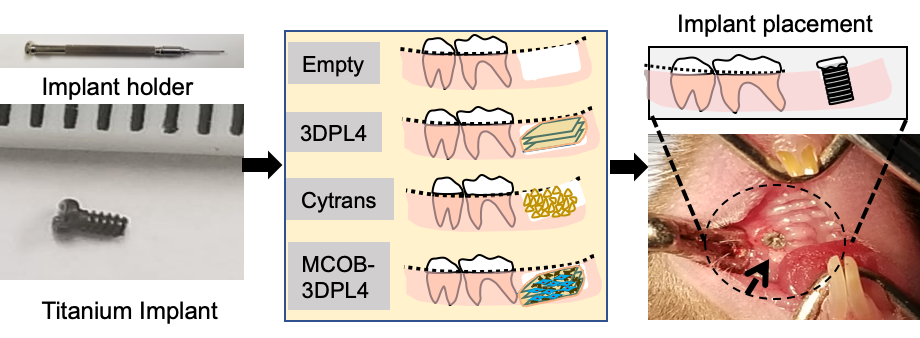


**Fig. S14. Schematic outline of the implant placement in regenerated bone.** Left panel: photographs of the implant holder and a titanium implant with a length of 1.5 mm and a diameter of 0.6 mm; central panel: representative experimental group in which the implant placed in regenerated bone (M1 area). Right panel: schematic (upper panel) and photograph (lower panel) of implant positioned in regenerated bone.


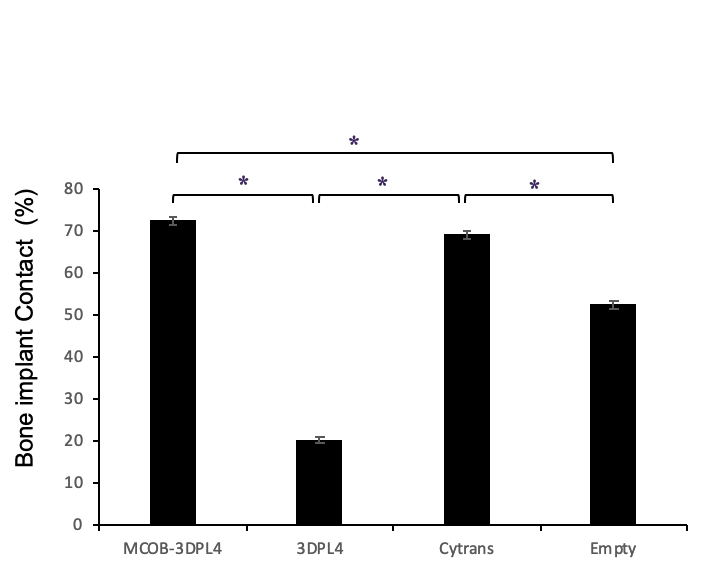


**Fig. S15.** Quantification of bone implant contact (BIC) ratios calculated from histological images at 4 weeks after implant placement.

**Supplementary Table 1**

Calculated average contact angle and surface roughness measurements of 3DPL and raw PLLA scaffold material.

|  | Average Contact angle (degree) | S.D. | Average Surface roughness[(Ra] (**μ**m) | S.D. |
| --- | --- | --- | --- | --- |
| 3DPL (pressed at 180^0^ C) | 69.7 | 1.5 | 0.101 | 0.012 |
| Raw PLLA (pressed at 180^0^ C) | 65.2 | 0.5 | 0.049 | 0.018 |

**Supplementary Table 2**

Sequences of the human- and mouse-specific gene primers used for quantitative real time RT-PCR.

Human primer sequences

| Gene | Sense | Antisense |
| --- | --- | --- |
| GAPDH | GTCAGTGGTGGACCTGACCT | GTCAGTGGTGGACCTGACCT |
| Osterix | CTGAAGAATGGGTGGGGAAGG | GGCCTCTGTCCTCCTAGCTC |
| Runx2 | GAAACTCAACAGATTAACTATCGTTTGC | GAATTTATCACAGATGGTCCCTAATGG |
| BSP | CGAATACACGGGCGTCAATG | GTAGCTGTACTCATCTTCATAGGC |
| Type I Collagen | CTCTCTCCACCTGCCTCTGG | CTTTGGGAAGTTGTCTCTGAAACC |

Mouse primer sequences

| GAPDH | CACTGAGCAAGAGAGGCCCTATCC | CCTAGGCCCCTCCTGTTATTATGG |
| --- | --- | --- |
| Osterix | TTTGCCAGTGCCTAGTTCCT | GGGAAAACGGCAAATAGGAT |
| Osteocalcin | TTGGTGCACACCTAGCAGAC | ACCTTATTGCCCTCCTGCTT |
| Runx2 | CGGTTCAGAGAGGTGGACTC | CTGCCTCTTGTCCCTTTCTG |

**Movie. S1**: Video demonstrating the implantation of MCOB-3DPL4 constructs into the mice alveolar bone defect.

**Movie. S2.** Video demonstrating implant placement in the regenerated bone.

**SI References**

**Reference:**

1. M. Aino, *et al.*, Isolation and characterization of the human immature osteoblast culture system from the alveolar bones of aged donors for bone regeneration therapy. *Expert Opinion on Biological Therapy* **14**, 1731–1744 (2014).

2. V. S. Venkataiah, *et al.*, Periodontal Regeneration by Allogeneic Transplantation of Adipose Tissue Derived Multi-Lineage Progenitor Stem Cells in vivo. *Sci Rep* **9**, 921 (2019).

3. E. Kato, K. Sakurai, M. Yamada, Periodontal-like gingival connective tissue attachment on titanium surface with nano-ordered spikes and pores created by alkali-heat treatment. *Dental Materials* **31**, e116–e130 (2015).
